# Dynamic gene expression response to altered gravity in human T cells

**DOI:** 10.1038/s41598-017-05580-x

**Published:** 2017-07-12

**Authors:** Cora S. Thiel, Swantje Hauschild, Andreas Huge, Svantje Tauber, Beatrice A. Lauber, Jennifer Polzer, Katrin Paulsen, Hartwin Lier, Frank Engelmann, Burkhard Schmitz, Andreas Schütte, Liliana E. Layer, Oliver Ullrich

**Affiliations:** 1 Institute of Anatomy, Faculty of Medicine, University of Zurich, Winterthurerstrasse 190, 8057 Zurich, Switzerland; 2 Department of Machine Design, Engineering Design and Product Development, Institute of Mechanical Engineering, Otto-von-Guericke-University Magdeburg, Universitätsplatz 2, 39106 Magdeburg, Germany; 3 Core Facility Genomic, Medical Faculty of Muenster, University of Muenster, Albert-Schweitzer-Campus 1, D3, Domagstrasse 3, 48149 Muenster, Germany; 4 KEK GmbH, Kemberger Str. 5, 06905 Bad Schmiedeberg, Germany; 5 Ernst-Abbe-Hochschule Jena, Carl-Zeiss-Promenade 2, 07745 Jena, Germany; 6 Airbus DS GmbH, Airbus-Allee 1, 28199 Bremen, Germany; 7 Zurich Center for Integrative Human Physiology (ZIHP), University of Zurich, Winterthurerstrasse 190, 8057 Zurich, Switzerland; 8 Institute of Space Life Sciences, School of Life Sciences, Beijing Institute of Technology, Beijing, 100081 China

## Abstract

We investigated the dynamics of immediate and initial gene expression response to different gravitational environments in human Jurkat T lymphocytic cells and compared expression profiles to identify potential gravity-regulated genes and adaptation processes. We used the Affymetrix GeneChip® Human Transcriptome Array 2.0 containing 44,699 protein coding genes and 22,829 non-protein coding genes and performed the experiments during a parabolic flight and a suborbital ballistic rocket mission to cross-validate gravity-regulated ﻿gene expression through independent research platforms and different sets of control experiments to exclude other factors than alteration of gravity. We found that gene expression in human T cells rapidly responded to altered gravity in the time frame of 20 s and 5 min. The initial response to microgravity involved mostly regulatory RNAs. We identified three gravity-regulated genes which could be cross-validated in both completely independent experiment missions: ATP6V1A/D, a vacuolar H + -ATPase (V-ATPase) responsible for acidification during bone resorption, IGHD3-3/IGHD3-10, diversity genes of the immunoglobulin heavy-chain locus participating in V(D)J recombination, and LINC00837, a long intergenic non-protein coding RNA. Due to the extensive and rapid alteration of gene expression associated with regulatory RNAs, we conclude that human cells are equipped with a robust and efficient adaptation potential when challenged with altered gravitational environments.

## Introduction

Due to their exceeding sensitivity to gravitational changes, cells of the human immune system represent an ideal model system to understand how gravity on Earth is required for normal cell function. *In vitro* studies with living human cells in microgravity, experiments on board of parabolic flights, suborbital or orbital flights, and ground-based facilities for simulated microgravity contributed a vast amount of knowledge bringing us closer to the potential primary cellular and molecular mechanisms behind the effects of altered gravity. Therefore, to analyze the immediate and initial response of gene expression to the different gravitational forces will help to identify primary gravity-regulated genes, while the investigation of time-effects in gene expression will contribute to an understanding of potential physiological fast adaptive responses to new gravity environments. For this reason, we investigated the time-course of the whole transcriptome response after alteration of the gravitational force in a parabolic flight and suborbital ballistic rocket experiment campaign. Our aim was to identify specific gravity-regulated genes by applying strict controls and cross-validation through two completely independent experiment missions.

Gene expression studies are indispensable for investigation and elucidation of molecular mechanisms, and whole genome expression profiles are offering the possibility to obtain an insight into networks and pathways of biomolecular interactions on a large-scale. Indeed, understanding the molecular and genetic basis of cellular response to altered gravity may provide important information for appropriate risk management, efficient monitoring and countermeasures against existing limiting factors for human health and performance in microgravity^1^. Moreover, analyzing the time-course of gene expression will provide crucial information about the existence and the extent of potential adaptation reactions in response to the alteration of the gravitational force that has been constant throughout the 4 billion years of Earth’s evolutionary history.

Since the early days of human spaceflight, an enhanced susceptibility to infections has been predicted for the Gemini missions^2^ and was observed during the Apollo missions^3^, where astronauts suffered from bacterial and viral infections. First evidence suggesting disturbed cellular function arose from investigations of lymphocytes from astronauts of the Soyuz and Skylab missions, that showed a considerably decreased response to mitogenic stimulation during and after flight^4, 5^. Then, during the first Spacelab mission, *in vitro* experiments confirmed a strongly impaired response of lymphocytes to proliferative stimuli under space conditions^6^. In this pioneering study more than thirty years ago, lymphocytes demonstrated not only less than 3% activation after Concanavalin A stimulation in microgravity compared to 1 g controls, but also an almost doubled proliferation rate when exposed to 10 g^6^. These findings provided first and clear evidence that cells are sensitive to gravity in principle, not only regarding lower but also regarding higher gravity compared to Earth. Many years later, another immune system disturbance was discovered exhibiting latent virus reactivation such as varicella zoster^7, 8^. Since the first evidences of a compromised immune system in space, sensitivity of cells of the human immune system to reduced gravity has been confirmed by numerous studies in real and simulated microgravity^9–15^. However, the mechanisms underlying gravity-regulated cellular responses are still unknown.

In the search for the molecular mechanisms, gene expression studies confirmed differential gene expression patterns in lymphocytes subjected to altered gravity: In peripheral blood lymphocytes, the expression of both IL2 and IL2 receptor alpha genes were significantly inhibited in simulated microgravity^16^, as well as PKC isoforms delta and epsilon^17^. A total of 91 genes with inhibited induction were found in microarray analysis after simulated microgravity, many of them early genes of T cell activation, regulated primarily by transcription factors NF-κB, CREB, ELK, AP-1, and STAT^18^. Another study identified 89 genes demonstrating altered expression in modeled microgravity, of which 79 were down-regulated and 10 were up-regulated^19^. Subsets of genes from that study comprised genes related to immune response, involved in signal transduction, related to apoptosis, transcription factor as well as structure-transport-binding protein genes, genes coding for proteins involved in protein folding and degradation, genes involved in tissue growth regulation or in control of different metabolic pathways, and finally histone, nucleotide binding, RNA-binding protein and DNA repair genes. Furthermore, microarray analysis revealed down-regulation of T cell activation genes, such as DAG kinase, human serine/threonine kinase, and tyrosine kinase, and up-regulation of HSP70 and IL4 receptor amongst others^20^. In total, 122 genes were detected in this study, affected by alterations of gravity. Rapid alteration of the cell cycle control protein p21 were observed in CD3/CD28-stimulated primary human T cells and in Jurkat T cells in real microgravity^21^. Yet another study observed inhibition of the Rel/NF-κB pathway by microgravity, and of the transcription of 47 genes, including immediate early genes in T cell activation^22^. In particular, transactivation of Rel/NF-κB, CREB, and SRF gene targets were down-regulated in microgravity, as well as transcription of REL itself. Results from these experiments indicate microgravity as causative factor for impaired T cell activation during spaceflight^22^. Recently, it was shown that also microRNA expression was altered during T cell activation under spaceflight conditions^23^. In that study, miR-21 was significantly up-regulated during early T cell activation in normal gravity, together with some of its biologically confirmed targets (EGR3, FASLG, BTG2, SPRY2, and TAGAP), while suppressed under microgravity. Further microarray analysis showed significant suppression of 85 other genes under microgravity conditions^23^. In Jurkat T cells, cDNA microarrays revealed altered cytoskeletal gene expression during spaceflight^24^. In that study, 11 cytoskeleton-related genes, including calponin, dynactin, tropomodulin, keratin 8, two myosins, an ankyrin EST, an actin-like protein, the cytoskeletal linker plectin, and a centriole-associated protein (C-NAP1) were up-regulated in microgravity compared with ground control cells; gelsolin precursor was down-regulated. Simulated microgravity revealed altered expression of microRNA in human lymphoblastoid TK6 cells including miR-150, miR-34a, miR-423-5p, miR-22, miR-141, miR-618, and miR-222^25^. This altered miRNA expression was confirmed to correlate with gene expression of several transcription factors including EGR2, ETS1, and REL. In murine cells from spleen, spaceflight effects on T lymphocyte distribution, function and gene expression revealed a decrease of IL2 and an increase of IL10, IFN-gamma, and MIP-1 alpha after activation with anti-CD3 antibody^26^. Analysis of cancer-related genes in murine thymus cells showed that the expression of 30 out of 84 genes was significantly affected by spaceflight: Birc5, Figf, Grb2, and Tert were up-regulated, while Fos, Ifnb1, Itgb3, Mmp9, Myc, Pdgfb, S100a4, Thbs, and Tnf were down-regulated^26^. Microarray analysis of spaceflown murine thymus tissue further revealed changes in gene expression regulating stress and glucocorticoid receptors, where Rbm3 was up-regulated, and Hsph110, Hsp90aa1, Cxcl10, Stip1, Fkbp4 were down-regulated^27^. QRT-PCR demonstrated additional gene expression alteration in other T cell related genes, including Ctla4, Ifna 2a (up-regulated) and Cd44 (down-regulated)^27^. Changes in mouse thymus and spleen were also investigated in a further study after return from the space shuttle STS-135 mission in which normal cell genes Il10, Il18bp, Il18r1, and Spp1 were found up-regulated, while Ccl7, Il6 were down-regulated, and cancer-related genes Casp8, Fgfr2, Figf, Hgf, IGF1, Itga4, Ncam1, Pdgfa, Pik3r1, Serpinb2, and Sykb were up-regulated as well, whereas Cdc25a, E2F1, Mmp9, and Myc showed decreased expression in thymus^28^. In spleen cells of that study, cancer-related gene Cdkn2a was found up-regulated by spaceflight, while Birc5, Casp8, Ctnnb1, Map2k1, Mdm2, Nfkb1, and Pdgfa showed less expression^28^. Another study with mouse spleenocytes revealed that spaceflight, as well as simulated microgravity caused significant reduction of key gene expression during early T cell activation^29^. Results demonstrated reduced expression of Il2, Il2 receptor alpha, IFN gamma, and TAGAP under real and simulated microgravity after T cell activation. Moreover, two new early T cell activation genes could be identified, Iigp1 and Slamf1, that showed reduced expression under microgravity as well. These findings indicated a significantly impaired function of the mouse immune system in spaceflight and in simulated microgravity^29^. However, the current knowledge is widely based on single data point analysis in studies with a great variability in design and system, which complicates the systematic validation across different models. Of course, this situation is mainly caused by the operational, technical and administrative constraints of spaceflight experiments. Isolated *in vitro* cell systems are representing very suitable models to study physiological and pathophysiological pathways induced by an altered gravitational environment, and are an important approach to understand the effect of space exploration on the immune system, as recommended also in the THESEUS (Towards Human Exploration of Space: A European Perspective, supported by the EU Seventh Framework Program for Research and Technology Development) study of the European Union for an integrated life sciences research roadmap in Europe^30, 31^.

The aim of our study was to identify gravity-sensitive gene expression in human cells through the combination of a parabolic flight and a suborbital ballistic rocket experiment and by applying strict controls to exclude other factors than alteration of gravity. Our approach also allowed the validation of gravity-regulated gene expression through two fully independent large scale research campaigns, providing a high level of confidence of any transcriptional changes independently identified in both campaigns. Furthermore, through the two measured time points of 20 s and 5 min real microgravity, the time course of gene expression changes and a possible adaptation response could be investigated. The two time points of real microgravity were provided by parabolic flights and a suborbital ballistic rocket experiment. During a parabolic flight, the aircraft (Airbus A300 ZERO-G) followed a Keplerian trajectory, described as an unpropelled body in an ideally frictionless space subjected to a centrally symmetric gravitational field. During this free-fall, the resultant of all forces acting on the aircraft other than gravity is nulled providing weightlessness within the aircraft. During the suborbital ballistic rocket flight of the TEXUS type rocket with a two-stage VSB-30 engine carrying the payload on top, the parabolic trajectory reaches an apogee between 250 and 300 km and provides more than 6 min microgravity, before re-entry and impact of the payload, slowed by a parachute. Gene expression profiles in the human Jurkat T cell line were previously analyzed after 48 hours microgravity during the Space Shuttle mission STS-95^24^. Therefore, we investigated differential gene expression in the Jurkat human T lymphocyte cell line during the 23rd parabolic flight campaign (PFC) and the TEXUS-51 suborbital rocket mission of the German Aerospace Center (DLR) to elucidate potential immediate adaptation and homeostasis responses to an altered gravity environment and to cross-validate the identification of gravity-regulated genes not only through independent experiments, but also through independent research platforms.

## Results

Aim of this study was to identify gravity-sensitive gene expression in a human cell line in a combination of parabolic flights with a suborbital ballistic rocket experiment and through the use of strict controls for excluding all possible other factors of influence. Our approach allowed the identification and validation of gravity-regulated gene expression through two fully independent large-scale research campaigns, in which sets of independent experiments were conducted. Therefore, transcriptional changes identified after both campaigns were characterized by a high level of evidence, not only due to several independent experiments, but also due to independent research platforms.

**Figure 1:**
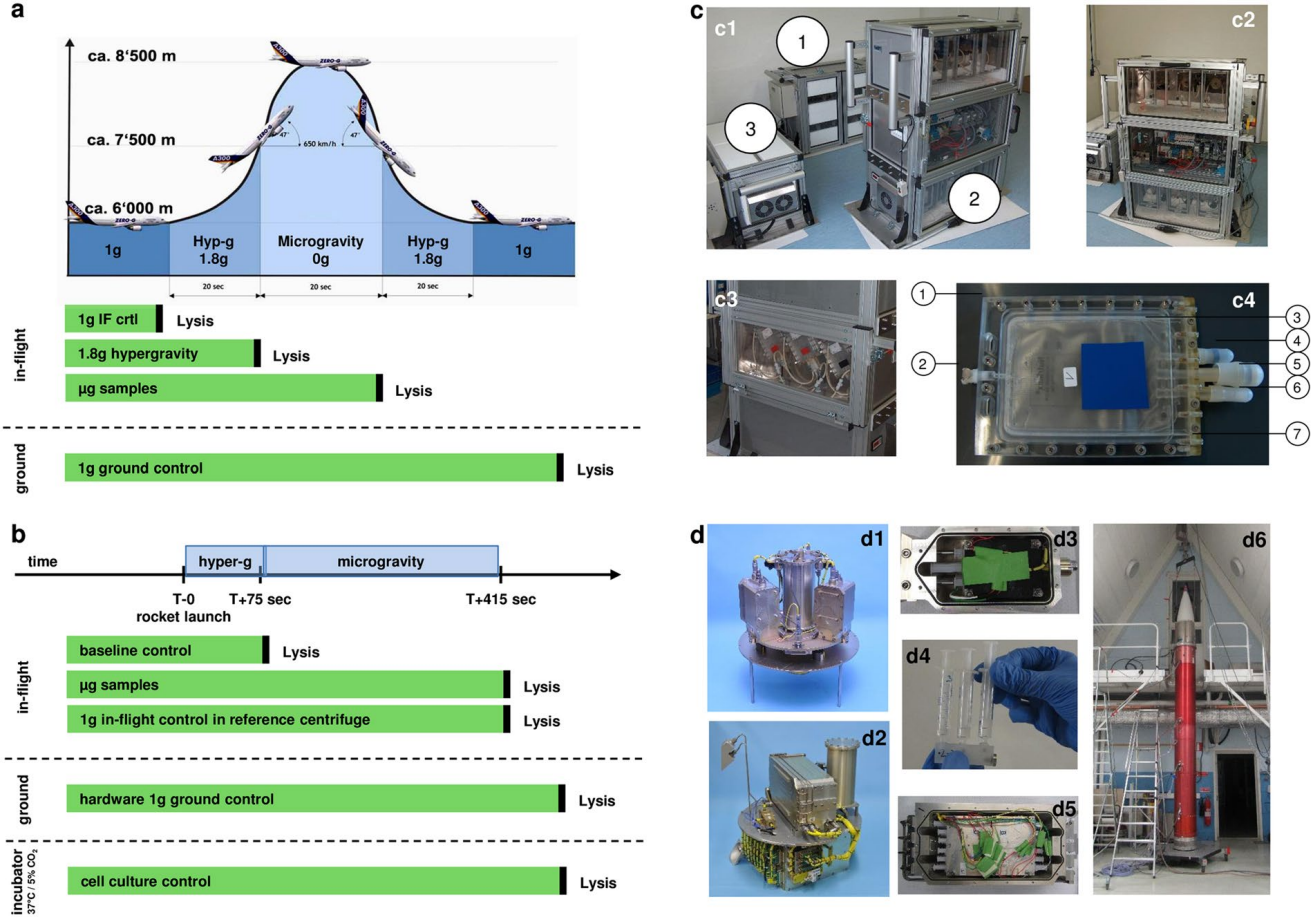
Experiment design of the parabolic flight (23^rd^ DLR PFC) and suborbital rocket (TEXUS-51) experiments. (**a**) During the 23^rd^ DLR PFC Jurkat T cells were analyzed before and during the first parabola. 1 g in-flight control (1 g IF ctrl) samples were lysed 5 min before the first parabola, 1.8 g hypergravity samples at the end of the first 1.8 g phase, and microgravity samples at the end of the first microgravity phase. After the flight, 1 g ground controls were performed in the experiment hardware inside the aircraft^66^. (**b**) During the TEXUS-51 sounding rocket mission Jurkat T cells were lysed at time point T + 75 s to monitor the hypergravity and vibration effects of the rocket launch. Microgravity samples and 1 g in-flight reference centrifuge control samples were lysed after 415 s post-launch. Additionally, hardware 1 g ground controls as well as cell culture controls were lysed post-flight approximately 15 min after the rocket launch. (**c**) In-flight experiment system for parabolic flights on board the Airbus A300 ZERO-G. c1 Experiment hardware structure which consists of an incubator rack to store the cell containers at 37 °C before the experiment (1), an experiment rack, in which all technical aggregates are accommodated for the execution of the experiment and where the living cells are processed during altered gravity (2), and a cooling rack to store all cell containers at 4 °C after the injection of the lysis solution until landing (3). c2 Structure of the working rack, rear side. In the upper third (4 °C) are three separate hose pumps which pump the lysis solution into the cell containers, controlled by the unit inside the middle third, which also carries all electrical connections and fuse elements. All liquids are sucked under exclusion of air. In the lower part (36.5 °C) are three separate hose pumps which pump the medium into the cell containers c3. Structure of the working rack, front side, waterproof working space with cell containers. c4. Double-walled, liquid-proof cell container. A maximum of 54 container can be accommodated during one flight. 1 = plastic container, 2 = air valve, 3 = internal sterile cell culture bag (Nutrimix, 0.25 l), 4 = connector 1 (medium), 5 = connector 2 (lysis buffer), 6 = connector 3 (port for filling of cells, performed pre-flight), 7 = plastic flange. (**d**) In-flight experiment system for the suborbital ballistic rocket flight of the TEXUS-51 payload. TEXUS consists of a VSB-30 engine (not shown) and of the payload structure (d6). Sets of three sterile syringes were filled with cell suspension, medium, and lysis buffer connected by a T-piece with small plugs at the outlet ports to prevent premature contact of the fluids (d4). The syringe systems are accommodated in tempered and vacuum-resistant containers (d3, d5) at the static (d2) or centrifuge (d1) position.

Furthermore, because of the two measured time points in real microgravity, 20 s and 5 min, the time course of gene expression changes and possible adaptation responses could be investigated. While most of the previous studies in real microgravity were end point experiments, our study allows to analyze the capability to react and adapt to a new gravitational environment. Adaptation effects were also in the scope of previous *in vivo* experiments determining blood cytokine concentrations in astronauts^32^. In our study, Jurkat T cells were cultivated and loaded into the hardware directly before the flight. During the parabolic flight, we used only the first parabola to analyze cells that never experienced hypergravity or microgravity before. Directly at the end of either condition, cells were lysed and the RNA was purified after landing. Cell samples lysed in-flight before the first parabola served as 1 g in-flight controls (Fig. 1a). In case of the suborbital rocket flight, Jurkat T cells were also lysed at the desired condition at the end of the hypergravity phase and at the end of the microgravity phase in parallel to the samples on the 1 g in-flight reference centrifuge (Fig. 1b). After the sample return, RNA was isolated. For each gravity condition at least 6 RNA samples were labeled and used for microarray hybridizations.

### Gene regulation response after cell cultivation in the flight hardware on board the Airbus A300 ZERO-G without gravitational changes

Table 1 displays the total number of transcripts that were significantly differentially expressed under altered gravity conditions during a parabolic flight. Surprisingly, 2432 transcripts were altered when comparing 1 g in-flight and the 1 g ground hardware control, clearly indicating that cell cultivation in the flight hardware on board the Airbus A300 ZERO-G induced a strong gene regulation response in the Jurkat T cell system. We therefore excluded transcripts differentially expressed in this comparison from further analyses.

**Table 1:** Differentially expressed transcripts in T cells during the 23^rd^ Parabolic Flight Campaign.

	1 g in-flight vs. H/W	hyp-g vs. 1 g in-flight	μg vs. 1 g in-flight	μg vs. hyp-g
**up-regulated transcripts**	**683**	**59**	**224**	**31**
coding:	495	21	164	21
non-coding:	188	38	60	10
**down-regulated transcripts**	**1749**	**47**	**55**	**12**
coding:	1181	42	47	11
non-coding:	568	5	8	1
**total number of differentially expressed transcripts**	**2432**	**106**	**279**	**43**

Number of significantly differentially expressed transcripts that were up or down-regulated in the respective comparison. H/W: hardware samples, hyp-g: hypergravity samples, μg: microgravity samples. Fold change ± 1.3, p < 0.05.

### Identification of rapid gravity-sensitive transcript regulation in the parabolic flight experiment

**Figure 2:**
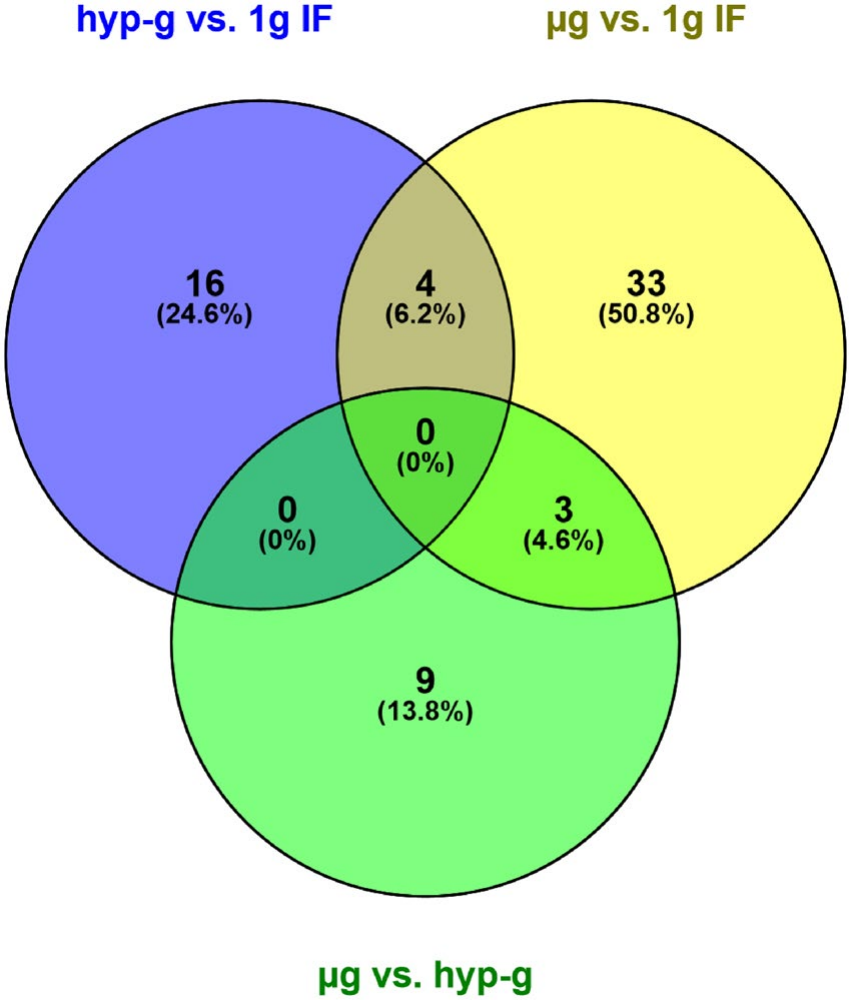
Venn diagram of overlapping and non-overlapping expression of significantly differentially expressed genes due to altered gravity conditions during the parabolic flight experiments. 1 g in-flight (1 g IF), 1.8 g hypergravity (hyp-g), and microgravity (µg). Venn diagram was constructed using Oliveros, J.C. (2007–2015) Venny, http://bioinfogp.cnb.csic.es/tools/venny/index.html.

**Figure 3:**
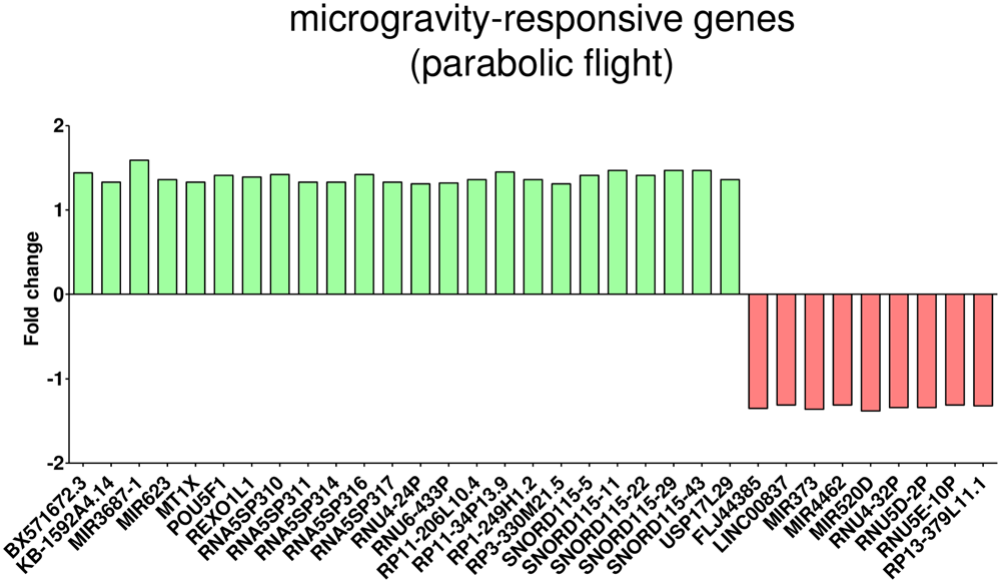
Microgravity-responsive gene expression (parabolic flight). Exposure of Jurkat T cells to 20 s of microgravity during the 23^rd^ DLR parabolic flight campaign led to 24 up and nine down-regulated genes when compared to the 1 g in-flight control (log2 values, p < 0.05).

**Figure 4:**
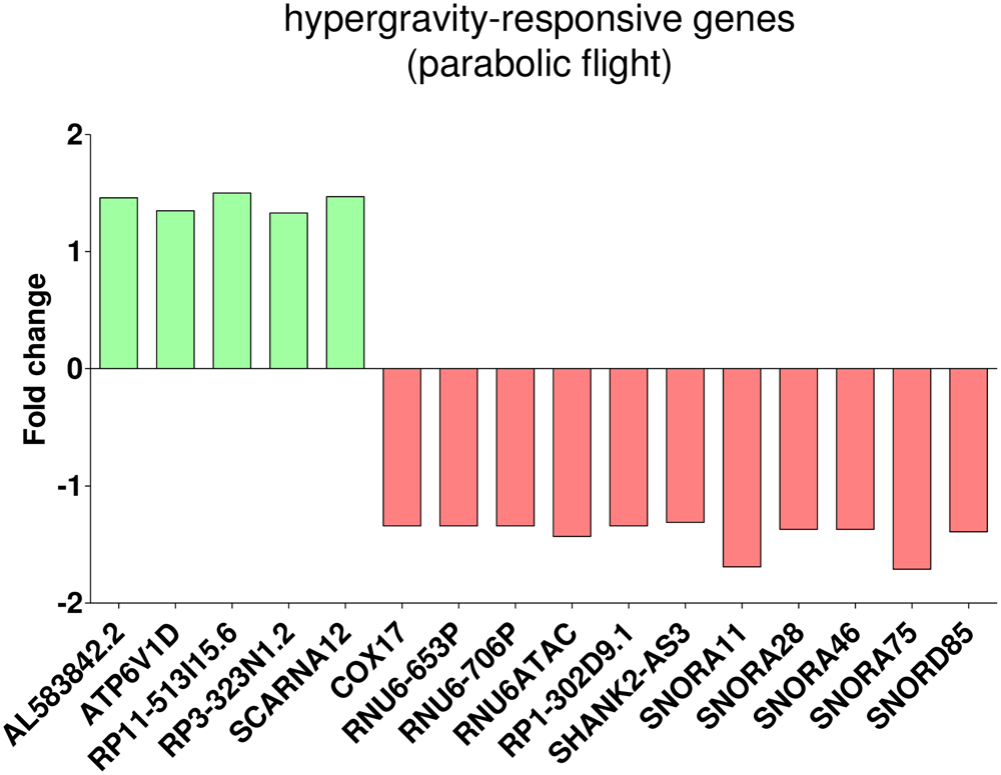
Hypergravity-responsive gene expression (parabolic flight). Exposure of Jurkat T cells to 20 s of hypergravity during the 23^rd^ DLR parabolic flight campaign led to five up and eleven down-regulated genes when compared to the 1 g in-flight control (log2 values, p < 0.05).

**Figure 5:**
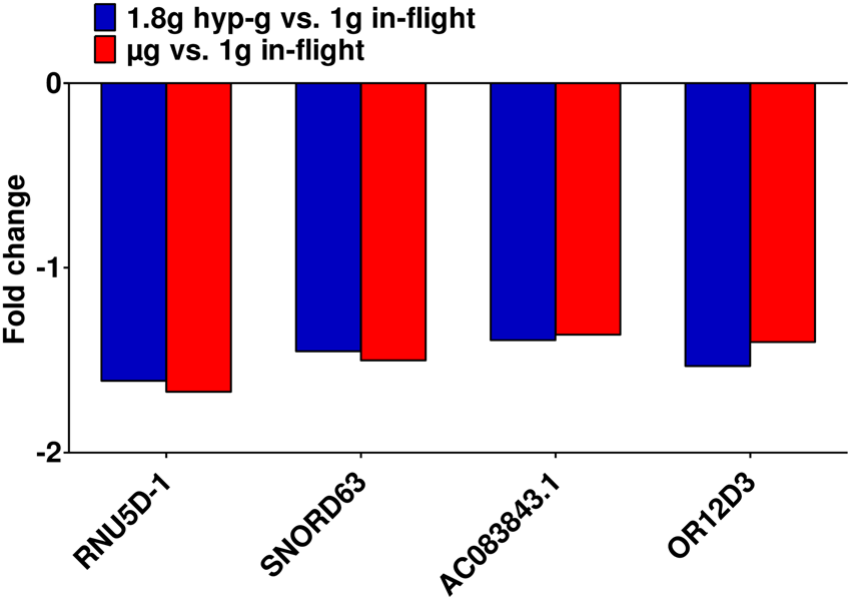
Gravity-responsive gene expression (parabolic flight). Expression level fold changes of genes in Jurkat T cells that are exclusively altered in both hypergravity (hyp-g) and microgravity (µg) when compared to 1 g in-flight controls during the 23^rd^ DLR parabolic flight campaign (log2 values, p < 0.05).

After the first hypergravity phase, 106 transcripts were differentially expressed and after the first microgravity phase, changes of 279 transcripts could be observed. When directly comparing microgravity and hypergravity, 43 changes were detected (Table 1). Within the annotated genes, only 23 genes were differentially expressed comparing hypergravity versus 1 g in-flight, and 44 genes comparing microgravity versus 1 g in-flight (Table 2). In order to exclude any possible prolonged gene expression changes induced by cultivation in the flight hardware during the flight, we excluded the 2432 genes that also showed changes in the comparison between 1 g in-flight versus 1 g hardware control (Table 3). In that way, by excluding all potential gene expression changes induced by cell cultivation during flight, we were able to unmask the effect of real microgravity and hypergravity. After this correction step, we found five genes up-regulated and 15 genes down-regulated in hypergravity. In microgravity, 40 genes were differentially expressed. In a further step, we focused on genes that were present in more than one comparison (Table 4 and Fig. 2). We identified four genes that were differentially regulated in hypergravity and in microgravity compared to 1 g in-flight (Figs 2 and 5). Figure 3 displays the 33 genes that are exclusively differentially expressed in microgravity compared to 1 g in-flight controls as shown in Table 4 and Fig. 2. Figure 4 shows the 16 genes differentially expressed solely in hypergravity compared to 1 g in-flight controls. Interestingly, most of the detected genes belong to the class of regulatory RNAs. Moreover, it could be observed that after 20 s of microgravity, the majority of genes was up-regulated, while after 20 s of hypergravity, the majority was down-regulated (Figs 3 and 4). The search for overlapping genes, differentially expressed in both microgravity and hypergravity and therefore supposed to be sensitive to altered gravity in general, revealed four candidates, among which are two small non-coding RNAs, RNUD-1 and SNORD63 and one long non-coding RNA, AC083843.1. Additionally, we could identify the G-protein coupled receptor OR12D3 (Fig. 5).

**Table 2:** Differentially expressed annotated genes in T cells during the 23^rd^ Parabolic Flight Campaign.

	1 g in-flight vs. H/W	hyp-g vs. 1 g in-flight	μg vs. 1 g in-flight	μg vs. hyp-g
**up-regulated genes**	362	6	28	12
**down-regulated genes**	832	17	16	3
**total number of differentially expressed genes**	**1194**	**23**	**44**	**15**

Number of annotated genes that were significantly differentially expressed in the respective comparison. H/W: hardware samples, hyp-g: hypergravity samples, μg: microgravity samples. Fold change ± 1.3, p < 0.05.

**Table 3:** Differentially expressed annotated genes in T cells during the 23^rd^ Parabolic Flight Campaign.

	hyp-g vs. 1 g in-flight	μg vs. 1 g in-flight	μg vs. hyp-g
**up-regulated genes**	5	26	9
**down-regulated genes**	15	14	3
**total number of differentially expressed genes**	**20**	**40**	**12**

Number of annotated significantly differentially expressed genes in the respective comparison after elimination of differentially expressed genes due to hardware effects (hardware 1 g ground control compared to the cell culture control) and 1 g in-flight effects (1 g in-flight control compared to the hardware 1 g ground control). hyp-g: hypergravity samples, μg: microgravity samples. Fold change ± 1.3, p < 0.05.

**Table 4:** Differentially expressed annotated genes in T cells during the 23^rd^ Parabolic Flight Campaign.

	hyp-g vs. 1 g in-flight	μg vs. 1 g in-flight	μg vs. hyp-g
**up-regulated genes**	5	24	7
**down-regulated genes**	11	9	2
**total number of differentially expressed genes**	**16**	**33**	**9**

Number of annotated significantly differentially expressed genes that are included in only one comparison after elimination of differentially expressed genes due to hardware effects (hardware 1 g ground control compared to the cell culture control) and 1 g in-flight effects (1 g in-flight control compared to the hardware 1 g ground control). hyp-g: hypergravity samples, μg: microgravity samples. Fold change ± 1.3, p < 0.05.

### Strong gene regulation response after cell cultivation in the flight hardware of the TEXUS-51 sounding rocket experiment

Cells are highly dynamic and can react transiently or permanently to stimuli in their environment. Therefore, we used a combined approach not only by intra-experimental comparisons, but also by inter-experiment comparison of parabolic flight and suborbital rocket experiment missions for validation of gravity-sensitive gene expression and for identification of potential adaptation processes in human T cells. The experiment performed on the TEXUS-51 suborbital rocket flight revealed a larger number of differentially expressed transcripts using the same microarray approach as for the parabolic flight experiment. A surprisingly high number of more than 22000 transcripts were differentially regulated when the hardware control was compared to the cell culture control (Table 5). These differentially expressed transcripts were excluded from further analyses. Therefore, we cannot exclude that some gravity-induced gene expression changes could be hidden by hardware-induced changes.

**Table 5:** Differentially expressed transcripts in T cells during the TEXUS-51 mission.

	H/W vs. CC	BL vs. H/W	BL vs. 1 g in-flight	µg vs. 1 g in-flight	µg vs. BL
**up-regulated transcripts**	**14581**	**2739**	**1732**	**1000**	**23**
coding:	10263	2027	1271	693	22
non-coding:	4318	712	461	307	1
**down-regulated transcripts**	**7649**	**955**	**588**	**873**	**22**
coding:	5698	743	410	614	19
non-coding:	1951	212	178	259	3
**total number of differentially expressed transcripts**	**22230**	**3694**	**2320**	**1873**	**45**

Number of significantly differentially expressed transcripts that were up or down-regulated in the respective comparison. CC: cell culture control samples, H/W: hardware 1 g ground control samples, BL: baseline control samples representing the influence of the rocket launch, µg: microgravity samples. Fold change ± 1.3, p < 0.05.

### Identification of gravity-sensitive transcript regulation in the suborbital rocket experiment and evidence for adaption responses of gene expression after 5 min altered gravity

**Figure 6:**
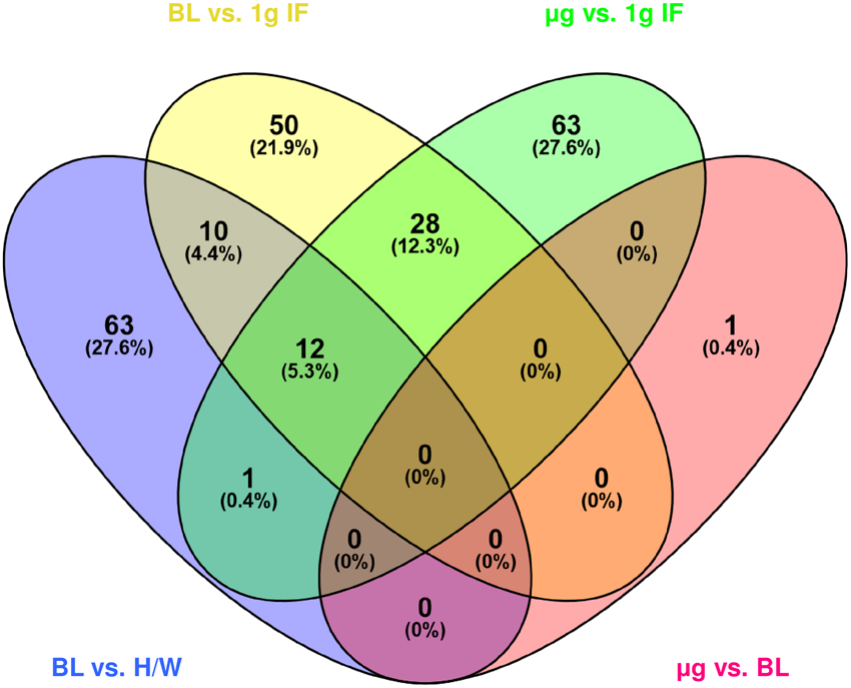
Venn diagram of overlapping and non-overlapping expression of significantly differentially expressed genes due to altered gravity conditions during the TEXUS-51 suborbital rocket flight. Hardware ground control (H/W), rocket launch baseline (BL), 1 g in-flight (1 g IF), and microgravity (µg). Venn diagram was constructed using Oliveros, J.C. (2007–2015) Venny, http://bioinfogp.cnb.csic.es/tools/venny/index.html.

**Figure 7:**
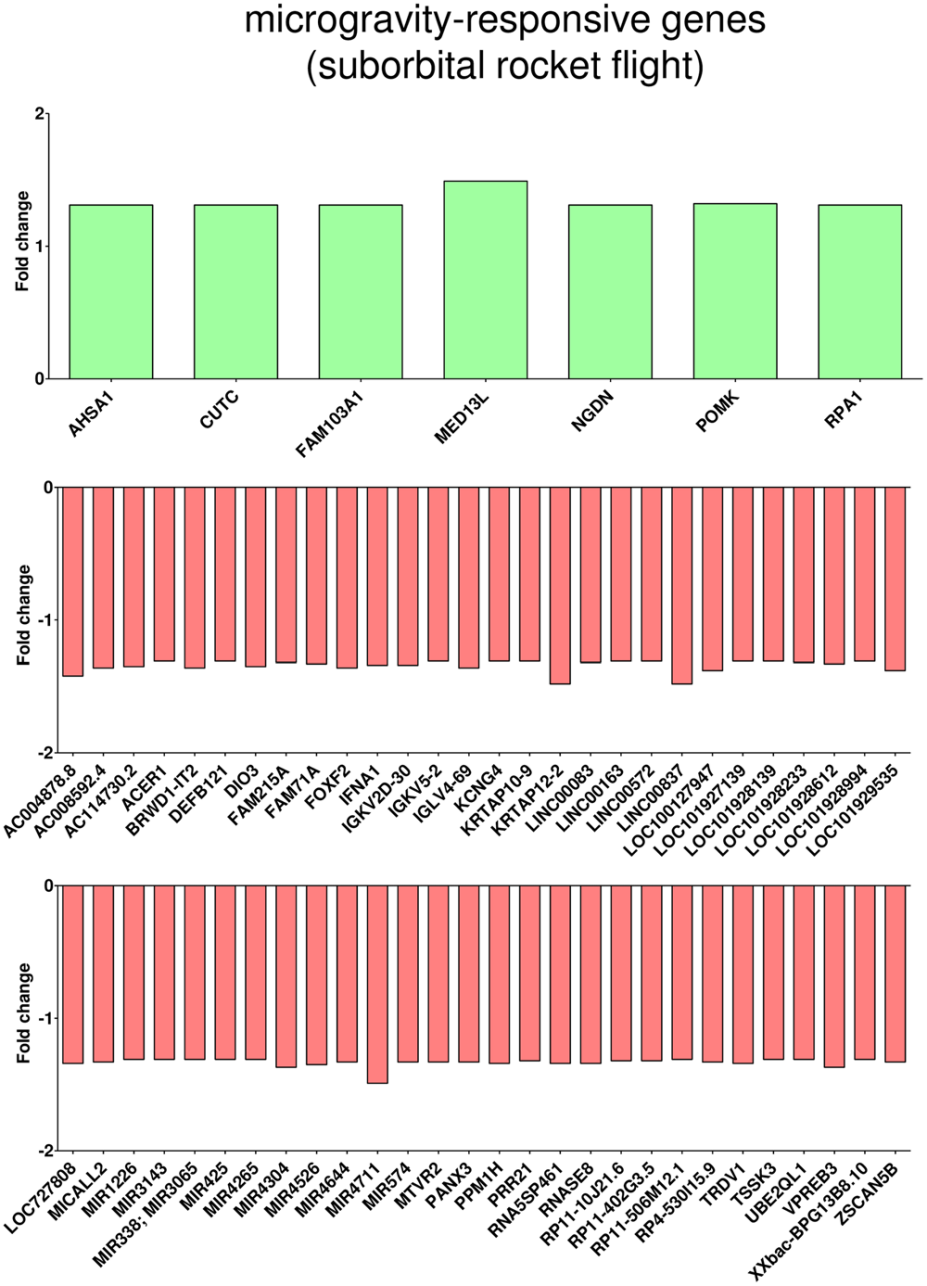
Microgravity-responsive gene expression (suborbital rocket flight). Exposure of Jurkat T cells to 5 min of microgravity during the TEXUS-51 suborbital rocket flight led to seven up and 56 down-regulated genes when compared to the 1 g in-flight control (log2 values, p < 0.05).

**Figure 8:**
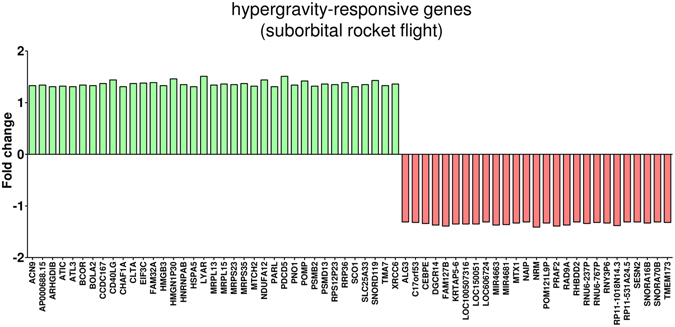
Hypergravity-responsive gene expression (suborbital rocket flight). Exposure of Jurkat T cells to hypergravity during the rocket launch of the TEXUS-51 sounding rocket led to 37 up and 27 down-regulated genes when compared to the 1 g in-flight control (log2 values, p < 0.05).

**Figure 9:**
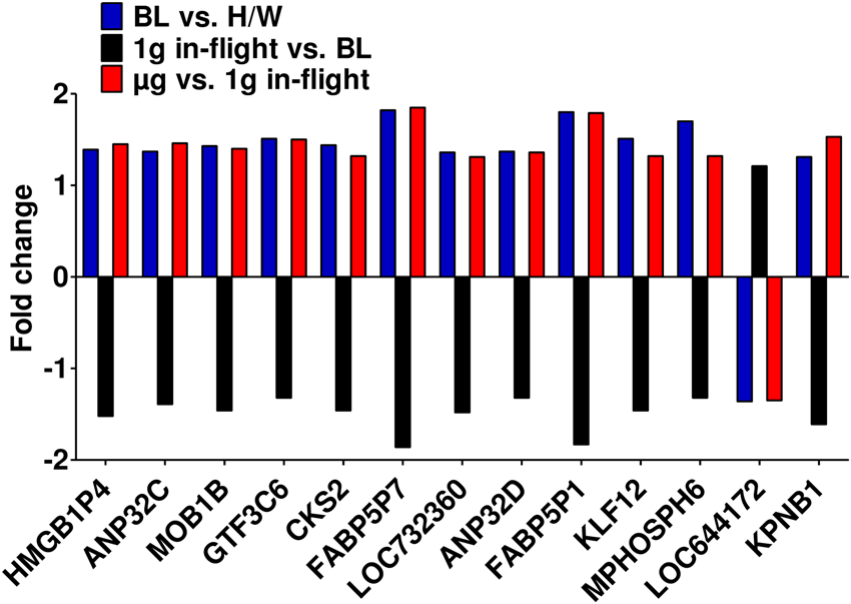
Gravity-responsive gene expression (suborbital rocket flight). Expression level fold changes of genes in Jurkat T cells that are exclusively altered during rocket launch (BL) when compared to the hardware ground control (H/W), and microgravity (µg) when compared to the 1 g in-flight control during the TEXUS-51 campaign (log2 values, p < 0.05).

We observed also high numbers of differentially regulated transcripts in altered gravity (Table 5) with approximately one half of annotated genes (Table 6). We again excluded all gene expression effects induced by the hardware, and finally identified 100 genes as differentially expressed in hypergravity and 104 genes in microgravity, compared to 1 g in-flight (Table 7). The Venn diagram in Fig. 6 shows the distribution of differentially expressed genes in all comparisons and provides an overview about genes that were changed only in one or in multiple comparisons. Those genes that are uniquely changed in one altered gravity condition are also depicted in Table 8. During the suborbital rocket flight, 7 up-regulated and 56 down-regulated genes were identified in microgravity (Fig. 7, Table 8), whereas in the preceding hypergravity phase (comparison between baseline versus hardware), 37 up-regulated and 26 down-regulated genes could be identified (Fig. 8, Table 8). In contrast, in the parabolic flight experiment, almost three times more genes were up-regulated than down-regulated in microgravity and twice as many genes were down-regulated than up-regulated in hypergravity (Figs 4 and 5), which represents the reverse situation. In Fig. 9 and Table 9, 13 genes are displayed that were differentially expressed in both microgravity and hypergravity during the TEXUS experiment. Among these genes, we found kinase activators important for the GPCR signaling, as well as a gene involved in Wnt signaling, a gene that is important for the nucleocytoplasmatic transport, two transcription factors, a gene that is involved in rRNA processing, and two tumor suppressor genes, and a cell cycle control gene. Interestingly, all genes adapted within 5 min 1 g on the reference centrifuge, revealed by the comparison between 1 g in-flight versus baseline (Fig. 9).

**Table 6:** Differentially expressed annotated genes in T cells during the TEXUS-51 mission.

	H/W vs. CC	BL vs. H/W	BL vs. 1 g in-flight	µg vs. 1 g in-flight	µg vs. BL
**up-regulated genes**	8244	1924	1178	640	5
**down-regulated genes**	4637	317	146	258	2
**total number of differentially expressed genes**	**12881**	**2241**	**1324**	**898**	**7**

Number of annotated genes that were significantly differentially expressed in the respective comparison. CC: cell culture control samples, H/W: hardware 1 g ground control samples, BL: baseline control samples representing the influence of the rocket launch, µg: microgravity samples. Fold change ± 1.3, p < 0.05.

**Table 7:** Differentially expressed annotated genes in T cells during the TEXUS-51 mission.

	BL vs. H/W	BL vs. 1 g in-flight	µg vs. 1 g in-flight	µg vs. BL
**up-regulated genes**	57	56	37	0
**down-regulated genes**	29	44	67	1
**total number of differentially expressed genes**	**86**	**100**	**104**	**1**

Number of annotated significantly differentially expressed genes in the respective comparison after elimination of differentially expressed genes due to hardware effects (hardware 1 g ground control compared to the cell culture control) and 1 g in-flight effects (1 g in-flight control compared to the hardware 1 g ground control). H/W: hardware 1 g ground control samples, BL: baseline control samples representing the influence of the rocket launch, µg: microgravity samples. Fold change ± 1.3, p < 0.05.

**Table 8:** Differentially expressed annotated genes in T cells during the TEXUS-51 mission.

	BL vs. H/W	BL vs. 1 g in-flight	µg vs. 1 g in-flight	µg vs. BL
**up-regulated genes**	37	18	7	0
**down-regulated genes**	26	32	56	1
**total number of differentially expressed genes**	**63**	**50**	**63**	**1**

Number of annotated significantly differentially expressed genes that are included in only one comparison after elimination of differentially expressed genes due to hardware effects (hardware 1 g ground control compared to the cell culture control) and 1 g in-flight effects (1 g in-flight control compared to the hardware 1 g ground control). H/W: hardware 1 g ground control samples, BL: baseline control samples representing the influence of the rocket launch, µg: microgravity samples. Fold change ± 1.3, p < 0.05.

**Table 9:** Gravity-responsive transcripts.

	Transcript	Function	Gene Ontology Molecular function	Gene Ontology Biological process	# in Fig. 13
**Parabolic flight experiment (23rd DLR PFC)**					
	RNU5D-1 RNA, U5D Small Nuclear 1	affiliated with the small nuclear ribonucleic acid, processing of pre-messenger RNA in the nucleus	**—**	**—**	8
	SNORD63 Small Nucleolar RNA, C/D Box 63	snoRNA	**—**	**—**	8
	AC083843.1	RNA Gene	**—**	**—**	8
	OR12D3 Olfactory Receptor Family 12 Subfamily D Member 3	G-protein-coupled receptors (GPCR), recognition and G protein-mediated transduction of extracellular signals	GO:0004888 transmembrane signaling receptor activity GO:0004930 G-protein coupled receptor activity GO:0004984 olfactory receptor activity	GO: 0007186 G-protein coupled receptor signaling pathway GO:0050907 detection of chemical stimulus involved in sensory perception GO:0050911 detection of chemical stimulus involved in sensory perception of smell	9
**Suborbital rocket experiment (TEXUS-51)**					
	GTF3C6 General Transcription Factor IIIC Subunit 6	GTFs assemble in a complex on the DNA promoter and recruit the RNA polymerase. GTF3C family proteins are essential for RNA polymerase III to make a number of small nuclear and cytoplasmic RNAs, including 5 S RNA tRNA, and adenovirus-associated (VA) RNA of both cellular and viral origin.	GO:0003677 contributes to DNA binding GO:0005515 protein binding	GO:0006351 transcription, DNA-templated GO:0006383 transcription from RNA polymerase III promoter GO:00427915 S class rRNA transcription from RNA polymerase III type 1 promoter GO:0042797 tRNA transcription from RNA polymerase III promoter	4
	KLF12 Kruppel Like Factor 12	Transcription Factor	GO:0000977 RNA polymerase II regulatory region sequence-specific DNA binding GO:0001227 transcription regulatory region sequence-specific binding GO:0003677 DNA binding GO:0003700 transcription factor activity, sequence-specific DNA binding GO:0003714 transcription corepressor activity	GO:0000122 negative regulation of transcription from RNA polymerase II promoter GO:0006351 transcription, DNA-templated GO:0006355 regulation of transcription, DNA-templated GO:0006357 regulation of transcription from RNA polymerase II promoter GO:0045944 positive regulation of transcription from RNA polymerase II promoter	4
	HMGB1P4 High Mobility Group Box 1 Pseudogene 4	**—**	**—**	**—**	**—**
	ANP32C Acidic Nuclear Phosphoprotein 32 Family Member C	tumor suppressor that can inhibit several types of cancers, including prostate and breast cancers	**—**	**—**	**7**
	ANP32D Acidic Nuclear Phosphoprotein 32 Family Member D	tumor suppressor that can inhibit several types of cancers, including prostate and breast cancers	**—**	**—**	**7**
	MOB1B MOB Kinase Activator 1B	related pathways are Signaling by GPCR and Hippo signaling pathway, related to kinase binding and kinase activator activity	GO:0005515 protein binding GO:0019209 kinase activator activity GO:0019900 kinase binding GO:0046872 metal ion binding	GO:0031952 regulation of protein autophosphorylation GO:0035329 hippo signaling GO:0042327 positive regulation of phosphorylation	1
	CKS2 CDC28 Protein Kinase Regulatory Subunit 2	CKS2 protein binds to the catalytic subunit of the cyclin dependent kinases inhibits the activation of cyclin A/CDK2 kinase, involved in the G1/S transition of the cell cycle	GO:0003682 chromatin binding GO:0005515 protein binding GO:0016538 cyclin-dependent protein serine/threonine kinase regulator activity GO:0019901 protein kinase binding GO:0042393 histone binding	GO:0006355 regulation of transcription, DNA-templated GO:0007127 meiosis I GO:0007346 regulation of mitotic cell cycle GO:0008283 cell proliferation GO:0044772 mitotic cell cycle phase transition	6
	FABP5P7 Fatty Acid Binding Protein 5 Pseudogene 7	Pseudogene	**—**	**—**	**—**
	FABP5P1 Fatty Acid Binding Protein 5 Pseudogene 1	Pseudogene	**—**	**—**	**—**
	LOC732360	Non coding RNA	**—**	**—**	8
	LOC644172 Mitogen-Activated Protein Kinase 8 Interacting Protein 1 Pseudogene	related to Wnt signaling pathway	**—**	**—**	2
	KPNB1 Karyopherin Subunit Beta 1	Nucleocytoplasmic transport, a member of the importin beta family	GO:0005515 protein binding GO:0008139 nuclear localization sequence binding GO:0008270 zinc ion binding GO:0008536 Ran GTPase binding GO:0008565 protein transporter activity	GO:0000059 protein import into nucleus, docking GO:0000060 protein import into nucleus, translocation GO:0006309 apoptotic DNA fragmentation GO:0006606 protein import into nucleus GO:0006607 NLS-bearing protein import into nucleus	3
	MPHOSPH6 M-Phase Phosphoprotein 6	involved in rRNA processing and gene expression, 3’-processing of the 7 S pre-RNA to the mature 5.8 S rRNA, might may play a role in recruiting the RNA exosome complex to pre-rRNA	GO:0003723 RNA binding GO:0005515 protein binding	GO:0000460 maturation of 5.8 S rRNA GO:0006364 rRNA processing	5
**Parabolic flight (23** ^**rd**^ **DLR PFC) and suborbital rocket (TEXUS-51) experiment**					
	ATP6V1A/D ATPase H + transporting V1 subunit	multisubunit enzyme that mediates acidification of eukaryotic intracellular organelles	GO:0005524 ATP binding GO:0046961 Protone-transporting ATPase activity, rotational mechanism	GO:0006810 transport GO:0008286 insulin receptor signaling pathway GO:0015991 ATP hydrolysis coupled proton transport GO:0016241 regulation of macroautophagy GO:0033572 transferrin transport	**—**
	LINC00837 Lonng Intergenic Non-Protein Coding RNA 837	Non-coding RNA class	**—**	**—**	**—**
	IGHD3-3/10 Immunoglobuline heavy diversity 3	V(D)J recombination in developing lymphocytes	**—**	**—**	**—**

Transcripts from parabolic flight (23^rd^ DLR parabolic flight campaign) and suborbital rocket experiments (TEXUS-51) that are differentially expressed in both, microgravity and hypergravity. Transcript names, annotated functions and gene ontology entries were adopted from the GeneCards encyclopedia (www.genecards.org). The numbers allocate the genes to the cellular processes and pathway components in Fig. 13.

### Most of the rapidly differentially expressed transcripts are regulatory RNAs

**Figure 10:**
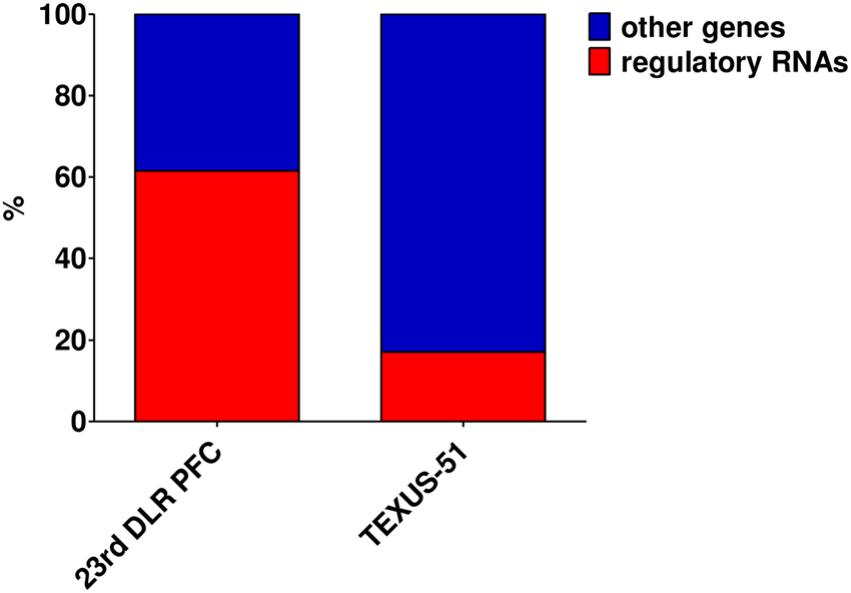
Regulatory RNAs. Percentage of regulatory RNAs that are significantly changed by hypergravity and/or microgravity during the 23^rd^ DLR parabolic flight campaign (23^rd^ DLR PFC), and the TEXUS-51 sounding rocket suborbital flight.

Further characterization of the identified differentially regulated genes, based on the annotated functions, showed a clear over-representation of regulatory RNAs after 20 s of microgravity and hypergravity during the parabolic flight: 61.5% of all differentially expressed transcripts were classified as regulatory RNAs. In contrast, during the hypergravity and microgravity phases of the suborbital rocket flight, only 17.1% of the up- or down-regulated genes belonged to the class of regulatory RNAs (Fig. 10).

### ATP6V1, LINC00837 and IGHD3 gene expression is gravity-regulated

**Figure 11:**
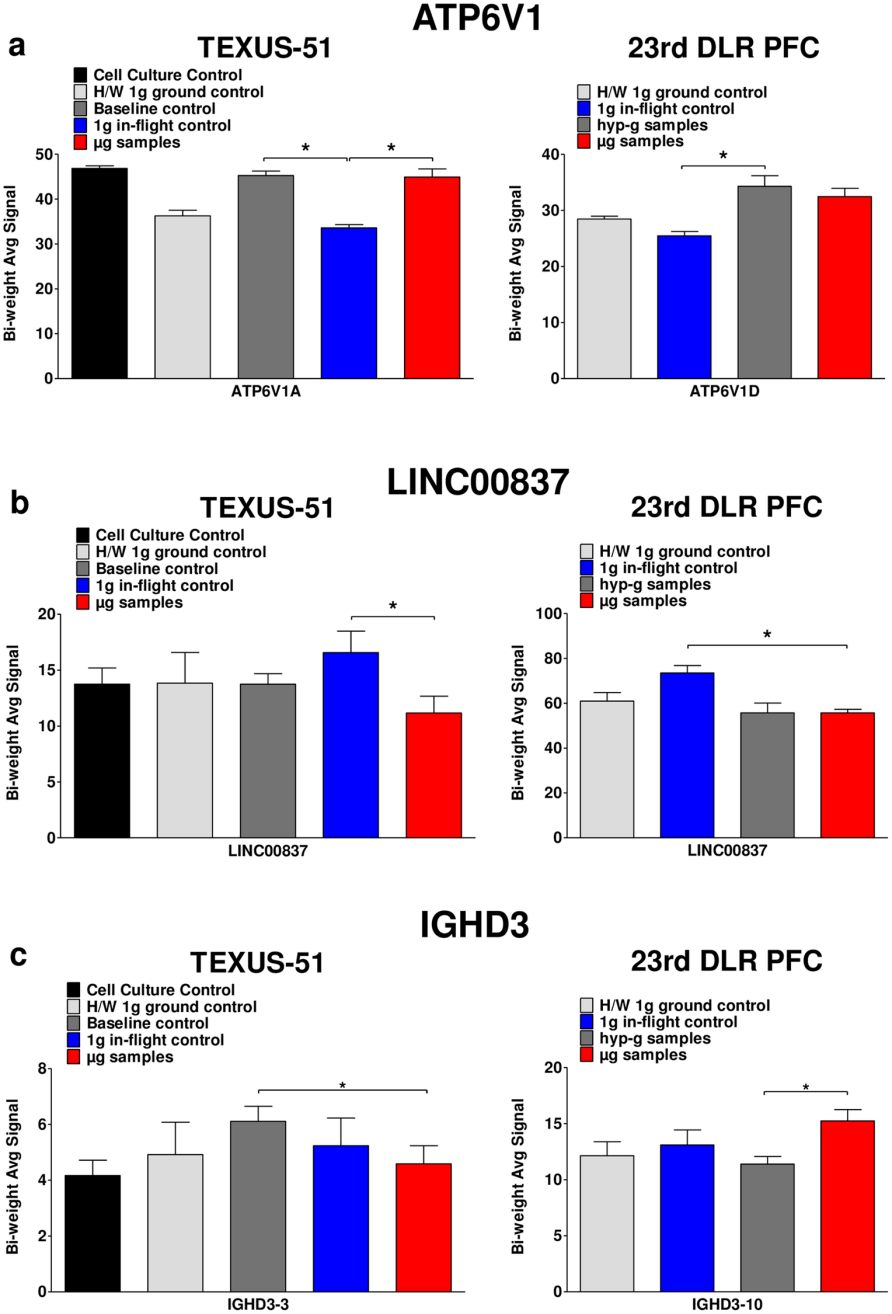
Gravity-regulated genes in human Jurkat T cells. Three genes were identified being differentially expressed under altered gravity conditions during parabolic flight and suborbital rocket flight. Tukey’s Biweight Function of linear values are demonstrated. (**a**) ATP6V1 encodes for a component of the H + vacuolar-type ATPase. After 20 s hypergravity, the D subunit of this enzyme is up-regulated compared to the 1 g in-flight control. After 75 s of hypergravity as well as after 5 min of microgravity, the A subunit of this protein is still significantly up-regulated. (**b**) LINC00837 is a Long Intergenic Non-Protein Coding RNA. This regulatory RNA is down-regulated after 20 s and 5 min of microgravity compared to 1 g in-flight. (**c**) IGHD3-3 and IGHD3-10 belong to the cluster of functional diversity (D) genes in the immunoglobulin (Ig) heavy chain locus on chromosome 14. IGHD3-10 is up-regulated in microgravity versus hypergravity during a parabolic flight, while during a sounding rocket flight, IGHD3-3 is down-regulated after 5 min of microgravity compared to the baseline control representing the hypergravity phase.

Finally, we analyzed the data to reveal transcripts that were differentially expressed after 20 s as well as after 5 min of altered gravity. We identified three transcripts that fulfilled this criterion (Fig. 11) and which were not altered in any of the strict control conditions: ATP6V1, LINC00837 and IGHD3. ATP6V1 is a multi-subunit enzyme that mediates acidification of eukaryotic intracellular organelles and is necessary for protein sorting, zymogen activation, receptor-mediated endocytosis, and synaptic vesicle proton gradient generation. The parabolic flight campaign results identified that the associated gene ATP6V1D was up-regulated in the comparison hypergravity versus 1 g in-flight control (Fig. 11a). After 75 s of hypergravity and 5 min of microgravity, subunit A of this enzyme remained up-regulated compared to the internal 1 g in-flight control. These data indicate that after up to 5 minutes of altered gravity, adaptation processes did not occur for this gene family. The second interesting gene that remained differentially expressed under altered gravity was the Long Intergenic Non-Protein Coding (LINC) RNA 837 (LINC00837). This regulatory RNA was down-regulated from 20 s up to 5 min of microgravity exposure (Fig. 11b). Thirdly, IGHD3-3 and IGHD3-10, belonging to the cluster of functional diversity (D) genes in the immunoglobulin (Ig) heavy chain locus on chromosome 14, remained differentially expressed in the inter-platform comparison. IGHD3-10 was up-regulated after 20 s microgravity compared to the hypergravity parabolic flight samples. IGHD3-3 was down-regulated after 5 min of microgravity compared to the baseline control, representing the hypergravity phase in this experimental setup (Fig. 11c).

**Figure 12:**
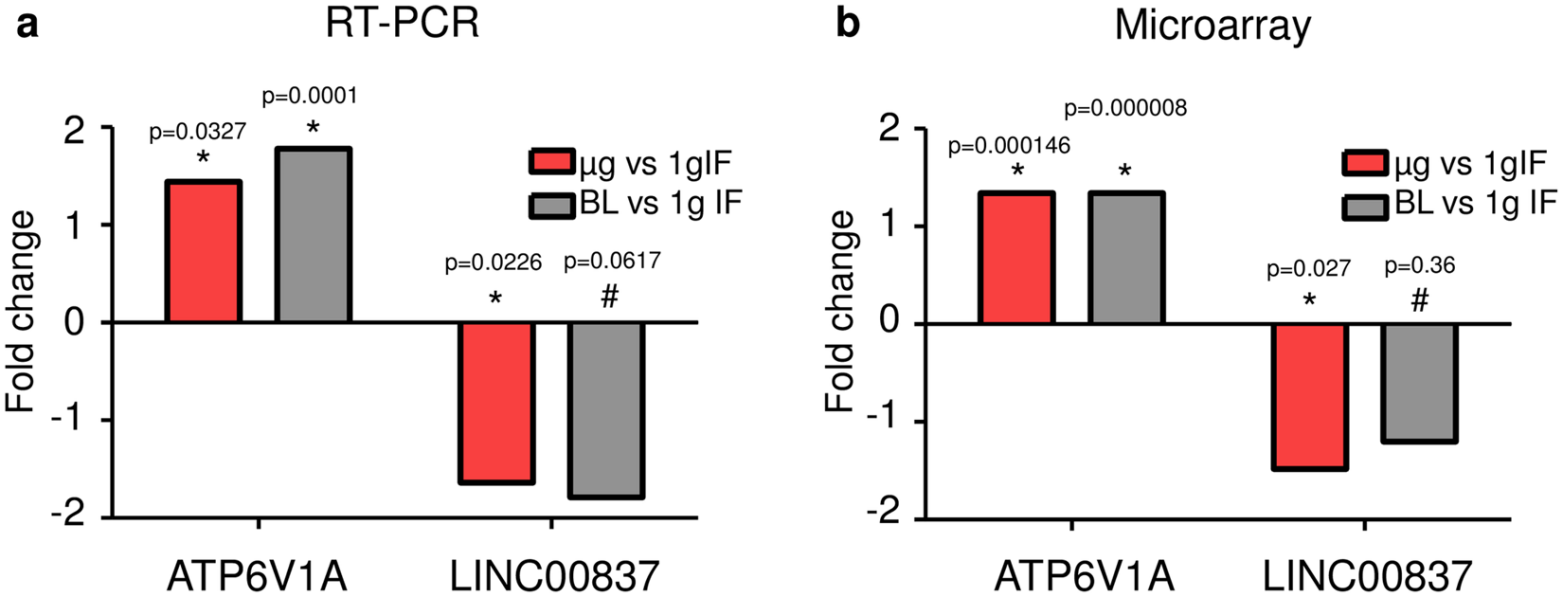
Quantitative real-time PCR validation of microarray expression data of ATP6V1A and LINC00837 for the TEXUS-51 suborbital rocket flight experiment. RNA samples used fo the microarray analysis were analyzed additionally by quantitative real time PCR as second independent method. (**a**) Microarray analysis: Significantly differential gene expression of ATP6V1A could be confirmed in microgravity (µg) versus 1 g in - flight (1 gIF) and in hypergravity (baseline BL) versus 1 g in - flight (1 gIF). Significantly differential gene expression of LINC00837 could be confirmed in microgravity (µg) versus 1g in - flight (1gIF). (**b**) Quantitative real time PCR analysis. Significant differential gene expression value of the same magnitude were detected in quantitative real-time PCR analysis. Statistical significance was tested by ANOVA for microarray results and by T-test in case of qRT-PCR, n = 9 for 1g in - flight (1gIF), n = 7 for baseline (BL) and n = 9 for ug.

Finally, we re-analyzed the RNA samples from both mission for the validation of gene expression data through a second independent analysis method, the quantitative real-time PCR. For the TEXUS-51 samples, quantitative real-time PCR analysis detected significant different gene expressions with values in the same magnitude as measured in the microarray analysis and therefore fully confirmed and validated the microarray data qualitatively and quantitatively. Therefore, two independent analysis methods revealed the up-regulation of ATP6V1A and down-regulation of LINC00837 in microgravity and in hypergravity compared to 1 g (Fig. 12). Although quantitative real time PCR analyses were also carried out for the parabolic flight samples according to the same standard methods and standardized PCR assays as for the TEXUS-51 samples, no significant changes in gene expression could be detected. Neither the up-regulation of ATP6V1D in microgravity and hypergravity compared to 1 g in flight (1.27- and 1.35-fold), nor the down-regulation of LINC00837 (1.31-fold) could be confirmed in real time PCR analysis due to the high variation of the measured values. Due to the very short 31 bp exon of IGHD3-3 and IGHD3-10, an appropriate primer pair design and quantitative PCR reaction was not possible, therefore preventing validation by quantitative real-time PCR as second independent analysis method. Therefore, gravity-dependent regulation of ATP6V1A and LINC00837 expression could be verified in independent experiments, on independent platforms and through independent analysis methods for the time point of 5 min, whereas gravity-dependent regulation of IGHD3 expression could be verified in independent experiments and on independent platforms.

## Discussion

We found changes in RNA abundance upon both microgravity and hypergravity compared to 1 g, clearly indicating a considerable gene expression reaction to altered gravity. The number of differentially regulated transcripts was higher after 5 min microgravity (1000 up-regulated and 873 down-regulated transcripts, Table 5) than after 20 s microgravity (224 up-regulated and 55 down-regulated transcripts, Table 1) compared to in-flight 1 g controls, respectively. Similar results were obtained when comparing differentially regulated transcripts in hypergravity versus 1 g in-flight controls: hypergravity during the TEXUS launch yielded 1732 up-regulated and 588 down-regulated transcripts (Table 5), while 20 s hypergravity during the parabolic flight resulted in 59 up-regulated and 47 down-regulated transcripts (Table 1). Amongst these transcripts, we detected a surprisingly high number of differentially expressed genes in the hardware sample control group of the PFC and of the TEXUS hardware, compared to standard cell culture conditions (Tables 1, 2, 5 and 6). After elimination of differentially expressed genes due to hardware effects, we identified 26 up-regulated and 14 down-regulated transcripts after 20 s of microgravity (Table 3) and 37 up-regulated and 67 down-regulated transcripts after 5 min microgravity (Table 7), compared to in-flight 1 g controls. After 20 s hypergravity, we identified 5 up-regulated and 15 down-regulated transcripts (Table 3) and after the hypergravity during the TEXUS ﻿flight 56 up-regulated and 44 down-regulated transcripts (Table 7). Therefore, gene expression is obviously severely altered after transfer into a new culture environment, although it consisted of approved biologically inert and sterile material, and although the cells had up to several hours to adapt to the new culture environment. Our findings support the mandatory requirement of strict ground controls in order to monitor the effect of the different culture conditions in flight hardware and to minimize or exclude the risk that adaptation responses to the new culture conditions are interfering with the microgravity experiment. Because our approach aimed at the validation of gravity-regulated gene expression with the highest possible level of evidence, we choose real microgravity experiments instead of ground-based simulations based on a vector-averaged Earth’s gravity environment, such as the Random Positioning Machine (RPM). Additionally, the RPM induces forces to the cell culture and makes it difficult to separate gravitational from fluid dynamic effects^33^. Finally, the high number of transcript changes observed in the flight hardware compared to other culture conditions on ground prevented us from the use of different hardware systems, which would be required for additional ground base platforms.

After excluding all potential non-gravity-related effects, we observed significant differential gene expression response between 1.3-fold up to almost 3-fold. Among the differentially expressed genes we found mRNA transcripts, long non-coding RNA (lncRNA), and several small RNAs as microRNA (miRNA), small nuclear RNA (snRNA), small nucleolar RNA (snoRNA), and small Cajal body RNA (scaRNA). The differential expression of miRNA in lymphocytic cells has already been reported earlier looking at activation^23^ and transcription factor expression^25^.

To further assess the degree of expression alteration, we compared our results to other studies, which investigated differential gene expression in non-activated lymphocytic cells under “stress conditions” other than gravitational changes: Scuba diving influenced the blood transcriptome with differential gene expression alterations between 1.5 up to 2-fold^34^. Another study described gene expression profiles of CD4 + lymphocytes at low and very low doses of ionizing radiation showing fold changes from 1.5 up to 2-fold in most cases, and in exceptional cases of up to more than 20-fold^35^. Gene expression profiles of human lymphocytes exposed to ^211^At α particles revealed expression changes of at least 2-fold up to over 10-fold^36^. These examples of other microarray studies on lymphocytic cells demonstrate that the values of fold changes observed in our study are in the usual range of that cell type. For comparison, we also found a study analyzing transcription of long non-coding RNA during CD4 + T cell development and activation reporting differential expression between non-activated and activated T cells in the range of 1.5 up to 3-fold that are consistent with the range of our data, too^37^. In contrast, if arbitrary cut off values are set too high (>1.5 to 2-fold), it is possible that genes representing important pathways are excluded^38^.

The findings of differential expression in altered gravity indicate that dysregulation and adaptation are processes that are not concluded within a couple of minutes. This hypothesis is corroborated by the discovery that the relative portion of differentially expressed regulatory RNAs is higher in short-term altered gravity (61.5%) compared to other RNAs, while it is strikingly lower in mid-term altered gravity (17.1%) (Fig. 10). Other studies confirm the differential expression of regulatory RNAs in altered gravity as well^18, 23, 25, 29, 38^. This points to active cellular reorganization and adaptation upon microgravity exposure rather than passive changes due to expression inhibition. This effect is later on attenuated during mid-term microgravity. In line with this hypothesis, we found more transcripts up than down-regulated after short-term microgravity (Fig. 3), and more transcripts down than up-regulated after mid-term microgravity (Fig. 7). A further gene annotation enrichment analysis using DAVID 6.8 was performed based on the genes presented in Fig. 5. Most likely due to the small number of genes, no specific pathway could be detected. However, as displayed in Table 9, the identified genes belong to RNA classes with regulatory or processing qualities, indicating that upon stimulus of altered gravity, cells react rapidly with a fast regulatory response to adapt to the new situation. Additionally, the detected regulatory processes are associated with a differentially expressed G-protein coupled receptor what might trigger further signal transduction processes.

Our experiments with non-activated T cells revealed different results than obtained for activated T lymphocytes regarding differential miRNA expression^22, 23^, or down-regulation of IL2 and IL2 receptor alpha^16, 29^, as well as of PKC isoforms delta and epsilon genes^17^. Furthermore, the differential expression of genes involved in early T cell activation after stimulation such as DAG kinase, HSP70 and IL4 receptor^20^, Rel/NF-κB, CREB, and SRF genes involved in the Rel/NF-κB pathway^22^ were not detected in non-activated T cells. By using non-stimulated T cells, we aimed at identifying the effects of gravity alteration on basic cellular homeostasis instead of investigating the activation processes. The combination of two platforms with different microgravity times allowed for the first time to elucidate basic cellular adaptation processes to the new environmental condition in altered gravity.

**Figure 13:**
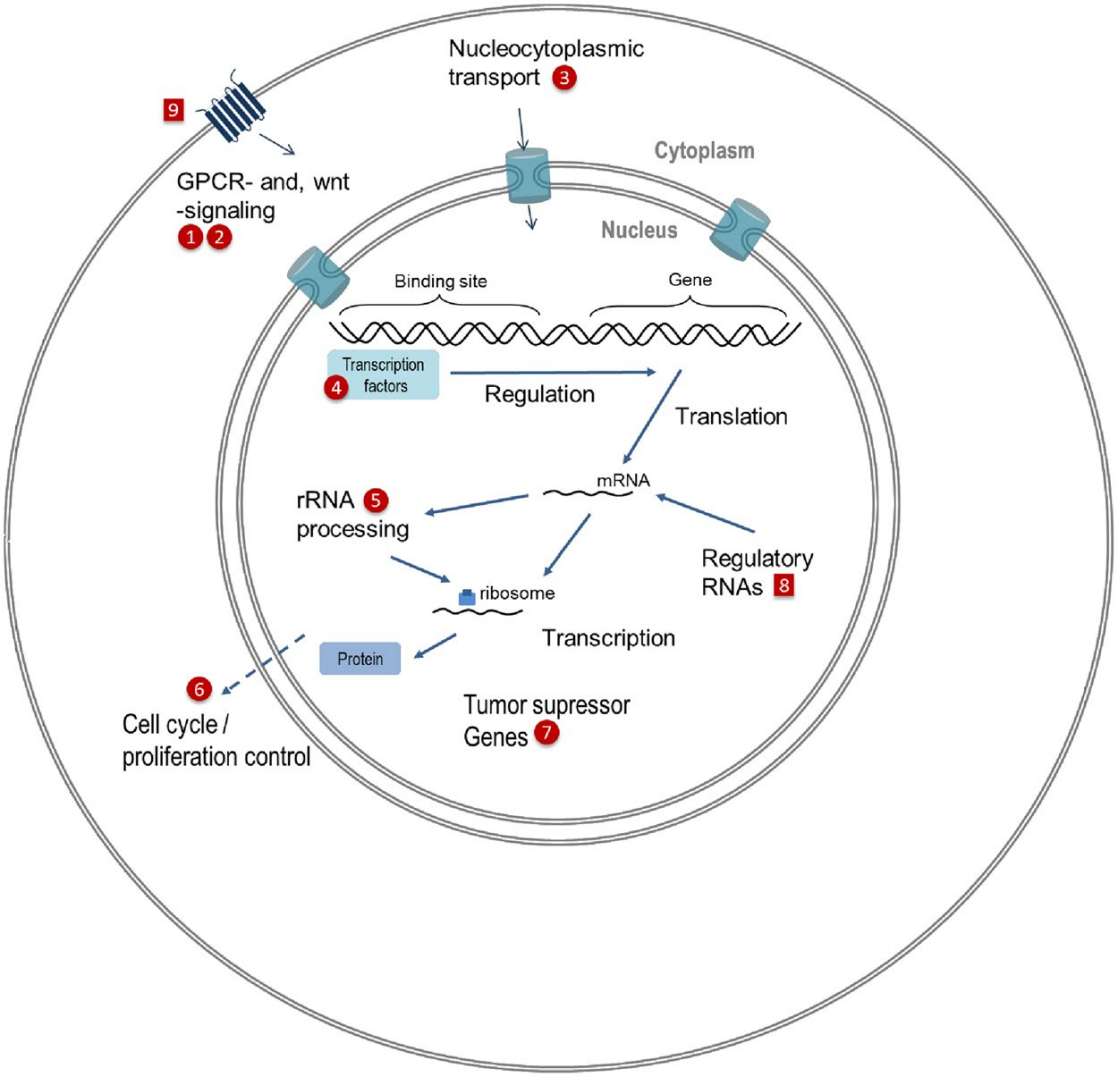
Hypothetical model of gravity-regulated cellular processes. Cellular processes and pathway components that were shown to be altered in microgravity and hypergravity during parabolic flight (numbers in squares) and sounding rocket flight (numbers in circles). The numbers represent the differentially expressed transcripts at their respective localization within the cell labeled with their participation in cellular processes. 1: MOB1B, MOB Kinase Activator 1B; 2: LOC644172, Mitogen-Activated Protein Kinase 8 Interacting Protein 1 Pseudogene; 3: KPNB1, Karyopherin Subunit Beta 1; 4: GTF3C6, General Transcription Factor IIIC Subunit 6; KLF12, Kruppel Like Factor 12; 5: MPHOSPH6, M-Phase Phosphoprotein 6; 6: CKS2, CDC28 Protein Kinase Regulatory Subunit 2; 7: ANP32C, Acidic Nuclear Phosphoprotein 32 Family Member C; ANP32D, Acidic Nuclear Phosphoprotein 32 Family Member D; 8: RNU5D-1, RNA U5D Small Nuclear 1; SNORD63, Small Nucleolar RNA C/D Box 63; AC083843.1; LOC732360; 9: OR12D3, Olfactory Receptor Family 12 Subfamily D Member 3.

During the TEXUS mission we observed substantially more differentially regulated genes in microgravity than during the parabolic flight experiments. This could either be due to the increase of time of altered gravity conditions the cells are exposed to or to higher mechanical stimulation during the preceding hypergravity phase (average TEXUS-51 acceleration is 5.1 g for 12 s for 1^st^ stage, and 6.7 g for 28 s for 2^nd^ stage compared to 22 s of 1.8 g during the parabolic maneuver). After exclusion of all genes that showed expression differences in the comparison of 1 g in-flight and 1 g hardware controls and differences in the comparison of 1 g hardware and standard cell culture controls, we identified 26 up and 14 down-regulated transcripts after the microgravity phase during the parabolic flight (Table 3), and 7 up and 56 down-regulated genes after microgravity during the sounding rocket flight (Table 8). The differentially expressed genes displayed in Table 8 and Fig. 9 indicate that the gravity-regulated genes identified in the TEXUS microarray analysis represent cellular processes downstream of the initially activated regulatory RNAs. We identified genes involved in inward-directed processes like GPCR and Wnt signaling, nucleocytoplasmic transport, transcription factors, rRNA processing, tumor suppressor genes and cell cycle regulator genes (Table 9, Fig. 13). A gene annotation enrichment analysis (DAVID 6.8) performed with these genes, revealed a significant (p-value 0.001) association with a histone binding function (GO:0042393) indicating also the inward-directed signal transduction with modifications in gene expression.

One of our aims was to identify gravity-sensitive gene expression, validated by two fully independent research platforms. As listed above, we found numerous genes that were dysregulated by either microgravity, hypergravity or both. After comparing the overall results of both independent platforms, parabolic flight and suborbital TEXUS flight, we were able to identify three genes, which were differentially expressed in both independent platforms: ATP6V1A/D, LINC00837, and IGHD3-3/IGHD3-10 (Table 9, Fig. 11).

Detailed sequence analysis of the above mentioned genes revealed that a further validation by quantitative real time PCR was possible only for ATP6V1A/D and LINC00837. IGHD3-3 and IGHD3-10 consist of only one short exon of 31 bp preventing a successful primer design to run a standard real time PCR assay. However, for ATP6V1A/D and LINC00837 validated commercial real time PCR assays are available. The analysis performed for the TEXUS-51 mission derived samples fully confirmed the microarray expression data. ATP6V1A was significantly up-regulated in microgravity compared to 1 g in-flight and in hypergravity compared to 1 g in-flight. LINC00837 was significantly down-regulated in microgravity compared to 1 g in-flight, as already detected in the microarray analysis (Fig. 12). Slight differences were observed between the microarray and real time PCR results concerning the magnitude of the fold change values most likely due to variations in individual sample processing (Fig. 12). In contrast to the TEXUS-51 samples, re-analysis of parabolic flight samples using quantitative real time PCR did not reveal significant differences, while we observed a higher variance in PCR analyses. The variance in the microarray data is probably lower due to parallel analysis and the internal controls present on every array. Microarray detection is probably the more sensitive and valid procedure when cellular reactions to mild stress factors like altered gravity are analyzed. It is well known that the quantitative values of microarrays and real time PCR are not interchangeable, while each of them has unique characteristics, making understanding the advantages and disadvantages of each technology useful in selecting the most appropriate technique for a determined purpose^39^.

Because validation of the microarray results by quantitative real-time PCR as second independent analysis was not possible for IGHD3-3 and IGHD3-10 due to the very short exon structure, gene expression differences of IGHD3-3 and IGHD3-10 could be validated through independent experiment missions and platforms, but not additionally through different analysis methods.

The vacuolar H+ −ATPase (V-ATPase) is composed of two multi-subunit domains, membrane embedded V0, and V1 associating with the cytosolic part of V0^40, 41^. V1 is made up of eight subunits (A–H), of which subunits A and B contribute to the catalytic ATP-binding sites, and D and F participate in forming the central stalk. The V-ATPase was long known to function as an electrogenic H + pump altering the pH of intracellular compartments. It thus controls enzyme activity, the dissociation of ligands from receptors, and the coupled transport of substrates across membranes^42–45^. More recently, however, the V-ATPase has been implicated in a variety of additional roles including fusogenicity, cytoskeletal tethering (anchorage site for the cytoskeleton), and metabolic sensing^46^. The association of the V-ATPase with actin was originally observed in osteoclasts^47, 48^, where it is responsible for acidification during bone resorption. It has been speculated that the V-ATPase promotes regulation of cytoplasmic G-actin pools, as well as crosslinking and stabilizing actin into filaments^49^. In our studies, we found V-ATPase subunits A and D up-regulated during hypergravity and microgravity (Fig. 11a). Interestingly, cytoskeletal degradation of Jurkat T cells in space environment has been reported earlier^50, 51^, which seems inconsistent with the finding of V-ATPase exerting a cytoskeleton stabilizing effect. However, it is conceivable that the cytoskeleton is degraded in microgravity by other means and the overexpression of subunits of the V-ATPase represents the cellular effort of counteracting this process. Vacuolar H + -ATPases (V-ATPases) are large multisubunit proton pumps that are required for housekeeping acidification of membrane-bound compartments in eukaryotic cells, including endosomes, lysosomes, compartments for uncoupling receptors and ligands, and elements of the Golgi apparatus^42^. Transcriptional regulation of the ubiquitous V-ATPase isoforms involves CpG islands containing multiple Sp1 and/or AP-2-like binding sites^52^, potentially subjected to cytosine methylation as form of epigenetic regulation^42^.

LINC00837 is a long intergenic non-protein coding RNA (lncRNA). LncRNAs were previously shown to be highly evolutionary conserved and to be functionally relevant^53^. LINC00837 was hypothesized to act as piRNA precursor^54^. Piwi-interacting RNAs (piRNAs) are a recently discovered class of small non-coding RNAs related to microRNAs, that were found to repress mobile element activity in animal germline, such as LINE-1 elements^55–58^. The human Long Interspersed Nuclear Element-1 (LINE-1) is a member of the group of autonomous non-LTR retrotransposons found in almost every eukaryotic genome. L1 elements generate copies of themselves by reverse transcription of an RNA intermediate and integration into the host genome. They are responsible for the generation of approximately 35% of the human genome, cover about 17% of it and represent the only group of active autonomous transposable elements in humans. L1 activity bears several risks for the integrity of the human genome, since the L1-encoded protein machinery generates DNA double-strand breaks, and is capable of conducting numerous genome-destabilizing effects, e.g. causing deletions at insertion sites, disrupting or rearranging coding sequences and deregulating transcription of functional host genes. On the other side, L1 elements have had and still exert a great impact on human genome structure and evolution by increasing the genome size and rearranging and modulating gene expression. We found LINC00837 significantly reduced after 20 s and 5 min microgravity (Fig. 11b) potentially facilitating LINE-1 activity. As LINE-1 elements exert great gene regulatory functions, it is possible that they are involved in gene expression changes and adaptation processes in microgravity.

IGHD3-3 and IGHD3-10 belong to the diversity genes of the immunoglobulin heavy-chain locus and participate in V(D)J recombination in developing lymphocytes during the early stages of T and B cell maturation. We found increased expression of IGHD3-10 after 20 s microgravity compared to hypergravity, and reduced IGHD3-3 expression after 5 min microgravity compared to hypergravity (Fig. 11c). Our *in vitro* observations are consistent with previous *in vivo* studies showing that Ig transcription^59^ and V(D)J recombination^60, 61^ are sensitive to altered gravity conditions. Therefore, the role of gravity for V(D)J recombination could be confirmed in independent research platforms as well as in independent animal experiments focusing on the B cell^60^ and T cell system^61^ and in our *in vitro* experiments using T cells. Jurkat T cells are a leukemic T-cell lymphoblast cell line, corresponding to an acute T cell leukemia, in which the presence of Ig transcripts was previously reported as a result of continuing V(D)J recombinase activity^62^. Consequently, expression of Ig transcripts in the Jurkat T cell line is not surprising, even if the function of these transcripts in Jurkat T cells is not known yet.

In our study, we investigated differentially regulated genes in non-activated human T lymphocytic cells in 20 s and 5 min microgravity and in hypergravity, and compared expression profiles to identify potential gravity-regulated genes and adaptation processes. We used the Affymetrix GeneChip® Human Transcriptome Array 2.0 containing 44699 protein coding genes and 22,829 non-protein coding genes, in sum 67,528 probes for gene transcripts. We identified a total number of 279 differentially expressed transcripts after 20 s of microgravity (parabolic flight) and 1873 differentially expressed transcripts after 5 min of microgravity (TEXUS), which corresponds to an expression change in 0.41% and 2.77% of detectable genes, respectively. After elimination of differentially expressed genes due to hardware effects, we identified a total number of 40 differentially expressed transcripts after 20 s microgravity (parabolic flight) and 104 differentially expressed transcripts after 5 min microgravity (TEXUS), which corresponds to an expression change in 0.06% and 0.15% of detectable genes, respectively. Interestingly, gene expression analysis in Jurkat T cells after 48 hours of exposure to microgravity aboard Space Shuttle mission STS-95 revealed only two percent either up- or down-regulated gene by 2-fold or greater compared to the ground controls^24^, which was in the same range as we already detected after 5 min microgravity. After 20 s microgravity, the transcript differences were detected mostly in regulatory RNAs, while after 5 min microgravity, we observed a progression in potential signal transduction cascades directed towards the nucleus where the gene expression pattern is altered (Fig. 13). The identified transcripts are involved in GPCR-signaling and wnt-signaling (MOB1B, LOC644172, OR12D3), nucleocytoplasmatic transport (KPNB1), transcription (GTF3C6), rRNA processing (MPHOSPH6), cell cycle and proliferation control (CKS2), tumor suppressor genes (ANP32C and ANP32D) and regulatory RNAs (RNU5D-1). Intriguingly, three differently expressed genes (MOB1B, KPNB1, ATP6V1A/D, Table 9) belong to gene families that were found hypermethylated in human lymphblastoid cells in simulated microgravity^63^. A recent publication reported that hypermethylation is not only involved in inhibition of gene expression, but rather in mediating gene expression than being exclusively responsible for inhibition or activation^64^.

The experiments of this study were performed in different very low gravity environments (10^−2^–10^−3^ g for parabolic flight experiments and 10^−4^ g for TEXUS experiments). Currently, it is unknown, in which extent different levels of very low gravity are transduced into a cellular response. Whereas a 2D clinostat study with 1F6 melanoma cells reported differences in guanylyl cyclase A mRNA expression in the range between 0.012–0.036 g^65^, the response of the oxidative burst reaction in NR8383 macrophages did not differ between the range of 10^−2^–10^−3^ g for parabolic flight experiments^66^ and the <10^−5^ g for the ISS experiment^67^. Additional, indications for a gravitational threshold between 0.3 g and 0.5 g were found^67^. Therefore, the current knowledge about biological effects of gravitational changes in the frame of the very low gravity environment is very limited.

In our study, we demonstrated that gene expression in human T cells rapidly responds to altered gravity in the time frame of 20 s and 5 min. Gravity-related gene expression was evidenced by rigorous control experiments and cross-validated in two completely independent experiment platforms, a parabolic flight and a sounding rocket flight. The initial response to microgravity involved mostly regulatory RNAs. Importantly, we detected extensive gene expression changes after transfer of the cells to a new culture environment, even after several hours. These findings raise serious questions about defining mandatory internal controls and implementing rigorous standardization of protocols into altered gravity experiments as well into 1 g experiments. For space experiments, sets of ground controls are mandatory to exclude the interference of cell culture adaptation processes with microgravity responses. Finally, we identified three gravity-regulated genes: ATP6V1A/D, a vacuolar H + -ATPase (V-ATPase) responsible for acidification during bone resorption, IGHD3−3/IGHD3-10, diversity genes of the immunoglobulin heavy-chain locus participating in V(D)J recombination, and LINC00837, a long intergenic non-protein coding RNA. Due to the extensive and rapid alteration of gene expression associated with regulatory RNAs, we conclude that human cells are equipped with a robust and efficient adaptation potential when challenged with altered gravitational environments.

## Material and Methods

### Cell culture

Jurkat cells (ATCC Clone E6-1, TIB152™)^68^ were used as a model cell line to analyze the differential gene expression under altered gravity conditions in human T cells. Jurkat T cells were cultivated in RPMI 1640 medium (Biochrom/Merck Millipore, Germany), supplemented with 10% fetal bovine serum (FBS Superior; Biochrom/Merck Millipore, Germany), 2 mM glutamine (low endotoxin; Biochrom, Germany) and 100 U/ml penicillin, as well as 100 µg/ml streptomycin (Biochrom, Germany). Cells were cultured with a density of 0.2 × 10^6^ cells/ml, and medium exchange was performed every 48 hours. Cells were centrifuged at 300 g for 5 min at room temperature, the supernatant was discarded, and the cell pellet was resuspended in fresh medium. An aliquot was taken, diluted with trypan blue solution and the vital cell number was counted. Cells were reseeded at a concentration of 0.2 × 10^6^ cells/ml in fresh medium.

### Parabolic flight experiment platform

Parabolic flights are an ideal platform to study initial and primary effects in mammalian cells and the associated rapid responsive molecular alterations excluding influences and interferences of secondary signal cascades. Parabolic flights offer a sequence of consecutive gravity conditions including 1 g, 1.8 g, and microgravity (µg) with a quality of 10^−2^ to 10^−3^ g (Fig. 1a). We designed and constructed an experimental system, which allows cell culture experiments during parabolic flights on board the Airbus A300 ZERO-G (reg. no. F-BUAD), which has been used already for different parabolic flight experiments^11, 13, 21^. Primary importance was placed on realizing the direct safety technique during the development activity. The experimental structure consists of three experiment racks (storage rack for cell culture containers before the experiments at 36.5 °C, cooling rack for storage of cell culture containers after cell lysis at 4 °C, and a working rack for handling and execution of the experiments) (Fig. 1c). The modular system is able to accommodate up to 54 cell culture containers (double containment) for each flight and allows storage of cell cultures until the start of the experiment, injection of a fluid (culture medium) at any defined time during the parabolic maneuver, and automatic injection of a second fluid (lysis buffer) after 20 s at the end of a defined gravity phase. Appropriate in-flight controls were obtained during the 1 g flight phase directly before the parabola. Injection of all fluids operates automatically and is pre-programmed, while exchange of cell culture containers and supervision of the experiment was performed manually. During the 23^rd^ DLR parabolic flight campaign (PFC), we investigated the gene expression in Jurkat T cells in microgravity and hypergravity (1.8 g) compared to in-flight 1 g. Experiments were only conducted during the first parabola to assure that detected differential gene expression levels were a result of the effect of gravitational change and not an accumulated long-term effect.

### Preparation and execution of the parabolic flight experiments

During the 23^rd^ DLR PFC, 1 × 10^7^ Jurkat T cells in 10 ml medium (RPMI 1640 supplemented with 100 U/ml penicillin, 100 µg/ml streptomycin, 2 mM glutamine and 10% FBS) were filled into 200 ml Nutrimix bags (B. Braun Melsungen, Germany) and transported from the home laboratory to the pre-flight preparation laboratories at the NOVESPACE premises in Bordeaux, France. After arrival, cells were stored at 36.5 °C overnight and used for the flight experiment on the following morning. 36.5 °C were chosen instead of 37 °C to rule out any thermic activation of the cells caused by regulatory oscillation of the storage rack. For the flight day, the Nutrimix bags were placed in a solid plastic housing to create a double containment that prevents spillage of fluids in the aircraft in case of leakage of the hardware system (Fig. 1c). Rapid lysis of Jurkat T cells in the respective gravity phase was achieved by fast injection of 5 volumes of RLT buffer (Qiagen, Germany) and mixing by inverting the samples three times immediately. The 1 g in-flight controls were performed 5 min before the first parabola, and the 1.8 g samples were lysed directly before the microgravity phase of the first parabola. The microgravity samples were lysed directly at the end of the microgravity phase of the first parabola. After landing, 1 g ground controls were performed immediately using the same hardware inside the aircraft. Figure 1a shows a schematic overview of the individual fixation time points for the samples of different gravity phases. Post-flight, all samples were directly transported to the on-site laboratory where total RNA was purified. In total, 24 samples were obtained: 6x 1 g ground controls, 6x 1 g in-flight controls, 6x 1.8 g and 6x µg.

### RNA isolation after the parabolic flight

After landing of the aircraft and transport of the samples to the laboratory facilities, the protective plastic housings were disassembled, the Nutrimix bags were gently agitated and the lysed cell solution was filled into a T75 straight neck cell culture flask. The cell solution was mixed for 10 s by vortexing and sheared by passing four times through an Ø 0.8 × 120 mm needle (B. Braun Melsungen, Germany) fitted to a sterile 50 ml syringe. 50 ml of absolute ethanol were added, and precipitates were resuspended by vigorous shaking. A Qiavac 24 plus vacuum system (Qiagen, Germany) was prepared by placing 24 valves and sterile connective pieces on the Qiavac 24 plus vacuum manifold and an RNA maxi column (Qiagen, Germany) was attached to each connective piece. The system was set to a vacuum level of −200 mbar, and the RNA maxi columns were loaded with the lysed cell suspensions. Subsequently, the valves were closed, and the RNA maxi columns were centrifuged at 3220 g for 3 min at room temperature and 15 ml of buffer RW1 (Qiagen, Germany) were carefully applied to the column to wash the membrane-bound RNA. After centrifugation at 3220 g for 7 min at room temperature, the flow through was discarded, and additional two washing steps were preformed with 10 ml RPE buffer (Qiagen, Germany) followed with centrifugation at 3220 g for 3 min and 10 min at room temperature, respectively. The column-bound RNA was eluted by application of 600 µl of pre-warmed RNase-free water (Qiagen, Germany), incubation for 1 min at room temperature and centrifugation for 4 min at 3220 g again at room temperature. The elution step was repeated with the first eluate, the column was centrifuged for 7 min at 3220 g, and the purified RNA was stored in a sterile 1 ml cryotube on dry ice. Finally, the extracted RNA was transported on dry ice and stored at −80 °C until the processing of the RNA for the microarray analysis.

### TEXUS-51 suborbital ballistic rocket experiment

TEXUS suborbital ballistic rockets consist of a two-stage VSB-30 rocket (S-30 solid rocket-stage engine with S-31 second-stage engine) and the payload (weight 390.4 kg, length 5083 mm). TEXUS-51 was launched on April 23rd, 2015 at 09:35 a.m. from the ESRANGE (European Space and Sounding Rocket Range) Space Center near Kiruna, Sweden, north of the Arctic Circle. During the ballistic suborbital flight, an altitude of 258 km and 369 s of microgravity with a quality of better than 10^−4^ g were achieved. Further parameters include: first stage peak thrust acceleration of 8.1 g at 2.4 s, mean thrust acceleration of 5.1 g, first stage burnout at 12.1 s, engine separation at 13.4 s, second stage peak thrust acceleration of 12.6 g at 34.9 s, mean thrust acceleration of 6.7 g, burnout at 43.2 s, spin at burn-out of 2.8 Hz, yo-yo despin at 56.0 s, engine separation at 59.0 s, maximum residual reentry deceleration of 13.9 g at 486.6 s, heat shield release at 7.4 g and 596.5 s, main parachute release at 2.4 g and 623.4 s, sink rate of 7.6 m/s, impact after 884 s at 68°34.6395′ N 21°09.1263′ E.

At ESRANGE, fully equipped laboratories enabled complete on-site preparation of the biological experiments, integration of the experiment into the payload platform 1 h before launch, and autonomous experiment execution in a programmed sequence. At the end of the free-fall period, the payload reentered the atmosphere and returned to the ground after parachute deployment at 5 km altitude and with a sink velocity of 8 m/s. A One helicopter immediately recovered the experimental unit. The payload was recovered by a second helicopter and returned to the launch site within 1.5 h after lift-off. The general experimental composition consists of multiple sets of three syringes, filled with cell suspension (human Jurkat T cells), cell culture medium, and lysis solution (Trizol LS). The syringe system is constructed to allow the addition of an active ingredient (e.g. for activation) to the cells prior to lysis or fixation. As this mission was planned to investigate the effect of gravity alterations in basal cell homeostasis, only cell culture medium was filled into the syringe for an active ingredient. All three syringes were connected by a T-piece, whereas small plugs at the outlet ports prevented premature contact of the fluids. The syringe systems were housed in a temperature controlled, vacuum-resistant container (Fig. 1d). The temperature controlled syringe systems were placed at microgravity positions inside the payload structure, as well as on a centrifuge, which generates 1 g gravitational force as reference (Fig. 1d). Before launch and during flight, syringes were activated by a pneumatic system at pre-set time points. Several pre-flight tests and development tests were conducted: Biocompatibility tests, chemical stability tests, culture medium optimization with regard to buffer systems and supplements, sterilization tests, viability tests, cell lysis tests (different lysis compounds and concentrations). The entire mission procedure was standardized and tested several times. Margins and possible holding times were determined. The experimental setup consisted of the baseline group (lysis at the end of the hypergravity phase and onset of microgravity), in-flight microgravity group (lysis after 5 min of microgravity and before reentry into the Earth’s atmosphere), 1 g in-flight reference group (lysis after 5 min of 1 g centrifugation and before reentry into the Earth’s atmosphere), 1 g ground control reference inside the experimental hardware, and cell culture controls (lysis on ground). Each experimental group consisted of at least seven samples. Cells, medium and lysis fluid (Trizol LS) syringes were prepared directly before the launch. All procedures started 7 hours before launch. The experimental containers were integrated into the payload structure by a “late access” port between 1:15 h and 0:45 h before launch. Sample temperature was maintained at 36.5 ± 0.5 °C until lysis. On landing and payload recovery, the experimental containers were immediately removed and returned to the ESRANGE laboratory for further processing. The cell suspension was transferred from the syringes into sterile plastic reaction tubes, and cells were homogenized with subsequent isolation of RNA. The purified RNA was stored and transported on dry ice or in liquid nitrogen, and analyzed afterwards by means of genome-wide Affymetrix Expression Arrays.

### Experimental preparation and integration for TEXUS-51

Jurkat T cells were cultured in the ESRANGE laboratories on site. Cells were cultivated with a density of 0.2 × 10^6^ cells/ml, and the medium was exchanged every 48 hours (see above). During the countdown phase, cells were visually inspected, harvested, the vital cell number was counted, and cells were pooled to a concentration of 5 × 10^7^ cells/ml. 0.5 ml of cells (i.e. 25 million cells) were filled in sterile 3 ml plastic syringes shortly before the handover to the launch team. Additionally, a second set of syringes was filled with 0.3 ml of cell culture medium and a third set with 1 ml Trizol LS (Life Technologies, Germany) per sample unit. The three syringes with small plugs at the outlet ports were mounted on a sterilized plastic T-block with a connecting tubing system. This experimental unit was finally integrated into the automatically operated experiment system. The experiment units were prepared and were kept at 36.5 ± 0.5 °C until the integration into the payload of the rocket in the late access phase. During the experimental run, firstly 0.3 ml of medium and secondly 1 ml of Trizol LS were injected to the cell suspension at defined time points to lyse the cells and preserve the current status of differential gene expression. The sequential injection of fluids was performed for the different sample groups as displayed in Fig. 1b. Directly before the µg phase, a set of samples was lysed at the time point of 75 s after launch (baseline, BL), representing the effect of the hypergravity, the spin and vibrations during the launch and rocket engine burn. Two further sets of samples were fixed at 375 s after launch, shortly before the end of the µg phase. One sample group was installed on an integrated 1 g centrifuge, while the other group represented the microgravity samples. Additionally, 1 g ground controls, as well as cell culture controls were kept on ground in the incubator analogously to the µg sample group. In total, 39 samples were obtained after the TEXUS-51 rocket flight: 7x 1 g ground cell culture controls, 7x 1 g hardware controls (H/W), 9× 1 g in-flight controls, 7x BL and 9x µg.

### RNA isolation after TEXUS-51 landing

Directly after landing, localization and recovery of the payload by helicopter, the experiment modules were dismantled and handed over for processing. The sample containing syringes were connected to a sterile 20 G needle (B. Braun Melsungen, Germany), the 1.8 ml of cell suspension were sheared three times and distributed equally in two 2.0 ml reaction tubes. 0.1 ml of chloroform (Sigma-Aldrich, Germany) were added, the homogenate was vortexed for 15 s and incubated for 5 min at room temperature before a 15 min centrifugation step at 11000 g and 4 °C. The upper phase within both 2.0 ml tubes was transferred into a 15 ml tube, and 4 ml of RLT buffer (Qiagen, Germany), as well as 3 ml of absolute ethanol were added, and the suspension was mixed. 4 ml of this solution were pipetted on an RNA Midi column (Qiagen, Germany), and centrifuged for 30 s at 3000 g and room temperature. The flow through was discarded and the residual 4 ml of RNA solution were loaded on the column and samples were centrifuged for 5 min at 3000 g at room temperature. Then, the columns were washed twice with 2.5 ml of RPE buffer (Qiagen, Germany), and centrifuged firstly for 2 min, and additionally for 5 min at 3000 g at room temperature. The RNA was eluted by the addition of pre-warmed 250 µl RNase-free water (Qiagen, Germany) to the column, incubation for 1 min at room temperature, and centrifugation for 3 min at 3000 g and room temperature. The flow through was loaded again onto the column, incubated for 1 min at room temperature, and centrifuged for 5 min at 3000 g and room temperature. The isolated RNA was transferred into sterile 1 ml cryotubes and stored and transported at −80 °C. After arrival in the home laboratory, samples were stored at −80 °C until the processing of the RNA for the microarray analysis.

### Sample processing and microarray analysis

Gene expression profiling was performed using Affymetrix GeneChip® Human Transcriptome Array 2.0 (Affymetrix United Kingdom Ltd., High Wycombe, United Kingdom) containing 44699 protein coding genes and 22829 non-protein coding genes. RNA quantity and quality were determined by measurement of concentration with absorbance at 260 and 280 nm (NanoDrop 2000c; Thermo, Fisher Scientific, Bonn, Germany) and by means of an Agilent 2100 Bioanalyzer with an RNA 6000 Nano kit and 2100 Expert software (version B.02.07) (all Agilent Technologies Deutschland GmbH, Boeblingen, Germany) at the Core Facility Genomics of the Medical Faculty Muenster (Muenster, Germany). Only high quality RNA with 260/280 nm ratios between 1.97 and 2.04 and RNA Integrity Numbers (RIN) > 8.2 was used for further microarray analysis. The fragmented and biotinylated DNA targets were prepared according to the standard Affymetrix WT PLUS Reagent Kit protocol (Affymetrix GeneChip® WT PLUS Reagent Kit, 902280) from 100 ng total RNA starting material and 5.5 µg cDNA intermediate product. DNA targets were hybridized for 17 h at 45 °C on GeneChip Human Transcriptome Arrays 2.0. GeneChips were washed and stained in the Affymetrix Fluidics Station 450 according to the standard GeneChip Expression Wash, Stain and Scan protocol (Affymetrix GeneChip Wash, Stain and Scan Kit, 900720). Subsequently, the GeneChips were scanned using the Affymetrix 3000 7 G scanner. For microarray data analysis, the Affymetrix Expression Console and Transcriptome Analysis Console was used. The robust multi-array (RMA) averaging method was applied for background correction, quantile normalization, and probe summarization. After background correction, the base2 logarithm of each background-corrected perfect-match intensity was obtained. These background-corrected and log-transformed perfect-match intensities were normalized using the quantile normalization method developed by Bolstad *et al*.^69^. In the quantile normalization method, the highest background-corrected and log-transformed perfect-match intensity on each GeneChip is determined. These values were averaged, and the individual values were replaced by the average. This process was repeated with what were originally the second highest background-corrected and log-transformed perfect-match intensities on each GeneChip, the third highest, etc. Gene expression differences were determined by applying an analysis of variance (ANOVA).

### Quantitative real-time PCR analysis

RNA samples were converted to cDNA with the High Capacity cDNA Reverse Transcription Kit (Thermo Scientific). Real-time PCR reactions were performed in triplicates using RT² SYBR® Green qPCR Mastermix (Qiagen) and RT² qPCR Primer Assay (Qiagen) on a CFX384 Real-Time PCR System (Bio-Rad, Germany), following manufacturer’s instructions. Dissociation curve analyses were carried out at the end of each run for PCR product verification. The data analysis using the comparative CT (delta delta CT) method^70^ was used for calculating relative quantitation of gene expression with the GAPDH housekeeping gene.

### Statistical analysis of selected genes

Genes of interest were identified, and the log2 values of the measured fluorescent intensities returned by the Affymetrix Expression Console were back calculated to linear values. Then, means of all values of the same gene generated by different probes were calculated. Subsequently, standard deviations were calculated for the means, and an unpaired t-test with Welch correction was performed using GraphPad Prism (T-Test, tails 2, type 3) to obtain statistical significance.

### Gene annotation enrichment analysis

The gene annotation enrichment analysis was carried out by using DAVID 6.8^71, 72^.

### Data Availability

The datasets generated during and/or analyzed during the current study are available in the GEO (Gene Expression Omnibus) repository (www.ncbi.nlm.nih.gov/projects/geo), accession no. GSE94256.

## References

[1] Comet, B. Limiting factors for human health and performance: microgravity and reduced gravity. *HUMEX-TN-002* Study on the survivability and adaptation of humans to long-duration interplanetary and planetary environments; Technical Note 2: Critical assessments of the limiting factors for human health and performance and recommendation of countermeasures (2001).

[2] Barry, C. A. & Catterson, A. D. Pre-Gemini Medical Predictions versus Gemini flight results in *Gemini Summary Conference*, *N*ASA-SP-138 (ed. NASA) 197–218, (NASA, United States, 1967).

[3] Nicogossian, A. E., Pool, S. L. & Uri, J. J. Historical perspectives in *Space physiology and medicine* (ed. Nicogossian, A. E., Huntoon, C. L. & Pool, S. L.) 3–49 (Lea & Febiger, 1993).

[4] Konstantinova IV, Antropova EN, Legen’kov VI, Zazhirei VD (1973). [Reactivity of lymphoid blood cells in the crew of “Soiuz-6”, “Soiuz-7” and “Soiuz-8” spacecraft before and after flight]. Kosm Biol Med.

[5] Kimzey, S. L. in *Biomed*ical R*es*ults *from Skylab* (eds R. S. Johnston & L. Dietlein) 249–283 (National Aeronautics and Space Administration, 1977).

[6] Cogoli A, Tschopp A, Fuchs-Bislin P (1984). Cell sensitivity to gravity. Science.

[7] Mehta SK (2004). Stress-induced subclinical reactivation of varicella zoster virus in astronauts. J Med Virol.

[8] Cohrs RJ, Mehta SK, Schmid DS, Gilden DH, Pierson DL (2008). Asymptomatic reactivation and shed of infectious varicella zoster virus in astronauts. J Med Virol.

[9] Crucian B, Stowe R, Quiriarte H, Pierson D, Sams C (2011). Monocyte phenotype and cytokine production profiles are dysregulated by short-duration spaceflight. Aviat Space Environ Med.

[10] Hughes-Fulford, M., Chang, T. & Li, C. F. In *Life in Space for Life on Earth*.

[11] Paulsen K (2015). Regulation of ICAM-1 in cells of the monocyte/macrophage system in microgravity. Biomed Res Int.

[12] Choukèr, A. & Ullrich, O. *The Immune System in Space: Are we prepared?*, (Springer International Publishing, 2016).

[13] Hauschild S (2014). T cell regulation in microgravity – The current knowledge from *in vitro* experiments conducted in space, parabolic flights and ground-based facilities. Acta Astronautica.

[14] Ullrich O, Huber K, Lang K (2008). Signal transduction in cells of the immune system in microgravity. Cell communication and signaling: CCS.

[15] Cubano LA, Maldonado HM (2006). Immune cells under altered gravity conditions. Bol Asoc Med P R.

[16] Walther I (1998). Simulated microgravity inhibits the genetic expression of interleukin-2 and its receptor in mitogen-activated T lymphocytes. FEBS Lett.

[17] Sundaresan A, Risin D, Pellis NR (2004). Modeled microgravity-induced protein kinase C isoform expression in human lymphocytes. J Appl Physiol (1985).

[18] Boonyaratanakornkit JB (2005). Key gravity-sensitive signaling pathways drive T cell activation. The FASEB journal.

[19] Ward NE, Pellis NR, Risin SA, Risin D (2006). Gene expression alterations in activated human T-cells induced by modeled microgravity. Journal of cellular biochemistry.

[20] Sundaresan A, Pellis NR (2009). Cellular and genetic adaptation in low-gravity environments. Ann N Y Acad Sci.

[21] Thiel CS (2012). Rapid alterations of cell cycle control proteins in human T lymphocytes in microgravity. Cell Commun Signal.

[22] Chang TT (2012). The Rel/NF-kappaB pathway and transcription of immediate early genes in T cell activation are inhibited by microgravity. J Leukoc Biol.

[23] Hughes-Fulford M, Chang TT, Martinez EM, Li C-F (2015). Spaceflight alters expression of microRNA during T-cell activation. The FASEB Journal.

[24] Lewis ML (2001). cDNA microarray reveals altered cytoskeletal gene expression in space-flown leukemic T lymphocytes (Jurkat). FASEB J.

[25] Mangala LS (2011). Effects of simulated microgravity on expression profile of microRNA in human lymphoblastoid cells. Journal of Biological Chemistry.

[26] Gridley DS (2009). Spaceflight effects on T lymphocyte distribution, function and gene expression. J Appl Physiol (1985).

[27] Lebsack TW (2010). Microarray analysis of spaceflown murine thymus tissue reveals changes in gene expression regulating stress and glucocorticoid receptors. Journal of cellular biochemistry.

[28] Gridley DS (2013). Changes in mouse thymus and spleen after return from the STS-135 mission in space. PloS one.

[29] Martinez EM, Yoshida MC, Candelario TLT, Hughes-Fulford M (2015). Spaceflight and simulated microgravity cause a significant reduction of key gene expression in early T-cell activation. American Journal of Physiology-Regulatory, Integrative and Comparative Physiology.

[30] Vernikos, J., Walter, N., Worms, J. C. & Blanc, S. THESEUS: The European research priorities for human exploration of space. *npj Microgravity* **2**, doi:10.1038/npjmgrav.2016.34 (2016). 10.1038/npjmgrav.2016.34 PMC5515530 28725741

[31] Frippiat, J.-P. *et al*. Towards human exploration of space: The THESEUS review series on immunology research priorities. *npj Microgravity* **2**, doi:10.1038/npjmgrav.2016.40 (2016). 10.1038/npjmgrav.2016.40 PMC5515533 28725745

[32] Crucian B (2015). Alterations in adaptive immunity persist during long-duration spaceflight. npj Microgravity.

[33] Wuest SL (2017). Fluid Dynamics Appearing during Simulated Microgravity Using Random Positioning Machines. PLoS ONE.

[34] Eftedal I (2013). Acute and potentially persistent effects of scuba diving on the blood transcriptome of experienced divers. Physiol Genomics.

[35] Nosel I, Vaurijoux A, Barquinero JF, Gruel G (2013). Characterization of gene expression profiles at low and very low doses of ionizing radiation. DNA repair.

[36] Turtoi A, Brown I, Schlager M, Schneeweiss FH (2010). Gene expression profile of human lymphocytes exposed to (211)At alpha particles. Radiat Res.

[37] Xia F (2014). Dynamic transcription of long non-coding RNA genes during CD4+ T cell development and activation. PloS one.

[38] Mukhopadhyay, S. *et al*. A systems biology pipeline identifies new immune and disease related molecular signatures and networks in human cells during microgravity exposure. *Scientific reports* **6** (2016). 10.1038/srep25975 PMC4868995 27185415

[39] Fujita A (2010). Comparison of gene expression profiles produced by CAGE, illumina microarray and real time RT-PCR. Genome Inform.

[40] Forgac M (2007). Vacuolar ATPases: rotary proton pumps in physiology and pathophysiology. Nature reviews. Molecular cell biology.

[41] Inoue T (2005). Structure and regulation of the V-ATPases. J Bioenerg Biomembr.

[42] Holliday LS (2014). Vacuolar H+ −ATPase: An Essential Multitasking Enzyme in Physiology and Pathophysiology. New Journal of Science.

[43] Huynh KK, Grinstein S (2007). Regulation of vacuolar pH and its modulation by some microbial species. Microbiol Mol Biol Rev.

[44] Nishi T, Forgac M (2002). The vacuolar (H+)-ATPases–nature’s most versatile proton pumps. Nature reviews. Molecular cell biology.

[45] Mellman I, Fuchs R, Helenius A (1986). Acidification of the endocytic and exocytic pathways. Annual review of biochemistry.

[46] Maxson ME, Grinstein S (2014). The vacuolar-type H(+)-ATPase at a glance - more than a proton pump. J Cell Sci.

[47] Lee BS, Gluck SL, Holliday LS (1999). Interaction between vacuolar H(+)-ATPase and microfilaments during osteoclast activation. The Journal of biological chemistry.

[48] Nakamura I (1997). Lack of vacuolar proton ATPase association with the cytoskeleton in osteoclasts of osteosclerotic (oc/oc) mice. FEBS Lett.

[49] Vitavska O, Merzendorfer H, Wieczorek H (2005). The V-ATPase subunit C binds to polymeric F-actin as well as to monomeric G-actin and induces cross-linking of actin filaments. The Journal of biological chemistry.

[50] Sciola L, Cogoli-Greuter M, Cogoli A, Spano A, Pippia P (1999). Influence of microgravity on mitogen binding and cytoskeleton in Jurkat cells. Adv Space Res.

[51] Lewis ML (1998). Spaceflight alters microtubules and increases apoptosis in human lymphocytes (Jurkat). FASEB J.

[52] Lee BS (2012). Regulation of V-ATPase expression in mammalian cells. Current Protein & Peptide Science.

[53] Guttman M (2009). Chromatin signature reveals over a thousand highly conserved large non-coding RNAs in mammals. Nature.

[54] Ha H (2014). A comprehensive analysis of piRNAs from adult human testis and their relationship with genes and mobile elements. BMC Genomics.

[55] Aravin AA (2009). Cytoplasmic compartmentalization of the fetal piRNA pathway in mice. PLoS genetics.

[56] Aravin AA (2008). A piRNA pathway primed by individual transposons is linked to de novo DNA methylation in mice. Molecular cell.

[57] Kuramochi-Miyagawa S (2008). DNA methylation of retrotransposon genes is regulated by Piwi family members MILI and MIWI2 in murine fetal testes. Genes & development.

[58] Carmell MA (2007). MIWI2 is essential for spermatogenesis and repression of transposons in the mouse male germline. Dev Cell.

[59] Huin-Schohn, C et al. Gravity changes during animal development affect IgM heavy-chain transcription and probably lymphopoiesis. *FASEB J*. **27****(****1****)**, 333-341, doi:10.1096/fj.12-217547. Epub 2012 Sep 19 (2013). 10.1096/fj.12-217547 22993194

[60] Bascove, M et al. Decrease in antibody somatic hypermutation frequency under extreme, extended spaceflight conditions. *FASEB J*. **25****(****9****)**, 2947-2955, doi:10.1096/fj.11-185215. Epub 2011 May 18 (2011). 10.1096/fj.11-185215 21593434

[61] Ghislin S (2015). Hypergravity exposure during gestation modifies the TCRβ repertoire of newborn mice. Sci. Rep.

[62] Szczepanski T (1999). Ig heavy chain gene rearrangements in T-cell acute lymphoblastic leukemia exhibit predominant DH6-19 and DH7-27 gene usage, can result in complete V-D-J rearrangements, and are rare in T-cell receptor alpha beta lineage. Blood.

[63] Chowdhury B (2016). A study of alterations in DNA epigenetic modifications (5mC and 5hmC) and gene expression influenced by simulated microgravity in human lymphoblastoid cells. PLoS One.

[64] Phillips T (2008). The role of methylation in gene expression. Nature Education.

[65] Eiermann P (2013). Adaptation of a 2-D Clinostat for Simulated Microgravity Experiments with Adherent Cells. Microgravity Science and Technology.

[66] Adrian A (2013). The oxidative burst reaction in mammalian cells depends on gravity. Cell Commun Signal.

[67] Thiel CS (2017). Rapid adaptation to microgravity in mammalian macrophage cells. Sci. Rep.

[68] Schneider U, Schwenk HU, Bornkamm G (1977). Characterization of EBV‐genome negative “null” and “T” cell lines derived from children with acute lymphoblastic leukemia and leukemic transformed non‐Hodgkin lymphoma. International journal of cancer.

[69] Bolstad BM, Irizarry RA, Åstrand M, Speed TP (2003). A comparison of normalization methods for high density oligonucleotide array data based on variance and bias. Bioinformatics.

[70] Livak KJ, Schmittgen TD (2001). Analysis of relative gene expression data using real-time quantitative PCR and the 2(-Delta Delta C(T)) Method. Methods.

[71] Huang DW, Sherman BT, Lempicki RA (2009). Systematic and integrative analysis of large gene lists using DAVID bioinformatics resources. Nature protocols.

[72] Huang DW, Sherman BT, Lempicki RA (2009). Bioinformatics enrichment tools: paths toward the comprehensive functional analysis of large gene lists. Nucleic acids research.

